# Optical Microscopy and Deep Learning for Absolute Quantification of Nanoparticles on a Macroscopic Scale and Estimating Their Number Concentration

**DOI:** 10.1021/acs.analchem.4c05555

**Published:** 2025-01-31

**Authors:** Antonín Hlaváček, Kateřina Uhrová, Julie Weisová, Hana Brožková, Naděžda Pizúrová

**Affiliations:** † Institute of Analytical Chemistry of the Czech Academy of Sciences, Brno 602 00, Czech Republic; ‡ Department of Chemistry, Faculty of Science, Masaryk University, Brno 602 00, Czech Republic; § Institute of Physics of Materials of the Czech Academy of Sciences, Brno 616 00, Czech Republic

## Abstract

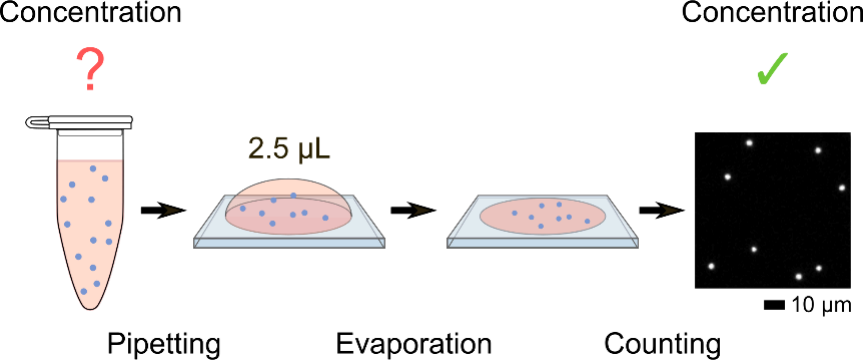

We present a simplistic and absolute method for estimating the number concentration of nanoparticles. Macroscopic volumes of a nanoparticle dispersion (several μL) are dropped on a glass surface and the solvent is evaporated. The optical microscope scans the entire surface of the dried droplet (several mm^2^), micrographs are stitched together (several tens), and all nanoparticles are counted (several thousand per droplet) by using an artificial neural network. We call this method evaporated volume analysis (EVA) because nanoparticles are counted after droplet volume evaporation. As a model, the concentration of ∼60 nm Tm^3+^-doped photon-upconversion nanoparticles coated in carboxylated silica shells is estimated with a combined relative standard uncertainty of 2.7%. Two reference methods provided comparable concentration values. A wider applicability is tested by imaging ∼80 nm Nile red-doped polystyrene and ∼90 nm silver nanoparticles. Theoretical limits of EVA such as the limit of detection, limit of quantification, and optimal working range are discussed.

Many new substances—nanoparticles—have been recently prepared and advanced research and technology. They also compose our environment and bodies where part of them is of natural origin and the other part is contributed by human activity. Besides others, the absolute quantification of nanoparticles and estimating their number concentrations (*i*.*e*. the number of nanoparticles in a given volume) are important for standardization, trading, studying nanoparticle properties, researching their role in the environment, and evaluating their toxicity.^1,2^ Available techniques for nanoparticle quantification measure either the ensemble property of nanoparticle dispersion or utilize nanoparticle counting.^1,2^ The counting is absolute and more straightforward.^1,2^ Two types of counting approaches—serial and parallel—can be recognized. In serial counting, the nanoparticles are detected individually when the dispersion streams through a detector. Optical detection,^3^ resistive pulse sensing,^4^ or single particle mass spectroscopy^5^ can be used. In a parallel setting, microscopy is used for imaging and counting large numbers of nanoparticles in a single micrograph. For instance, optical microscopy was used for counting and tracking single nanoparticles in a free dispersion.^6^

However, these methods are limited by short detection times, which prevent us from counting small nanoparticles (∼30 nm) with generally low signals.^6,7^ In serial counting, the detection time is limited by a need for sufficiently fast flow and diffusion of nanoparticles imposes limits in single-particle tracking. The problem of moving nanoparticles was solved by their embedding in gels^8^ or resins.^9^ However, the detection is still limited by a background signal^7^ from the gel-enclosed solvent, gel matrix, or resin, and the complexity of instrumentation.^8,9^ To overcome these limitations, specialized sampling approaches are developed when the nanoparticles are immobilized on a suitable substrate and the solvent is removed traceably. For instance, a nanopipette was designed for transmission electron microscopy (TEM),^10^ and anisotropically collapsing gels were used for optical microscopy.^11^

Here, we present a simplistic approach (Scheme 1). A macroscopic volume of nanoparticle dispersion is dropped on a glass surface and the solvent is freely evaporated. An optical microscope scans the dried droplet, and all nanoparticles are counted. We call this method evaporated volume analysis (EVA) because all nanoparticles are counted after droplet volume evaporation. Indeed, the EVA is not a completely new technique and was used either for larger particles with specific imaging signals (*e*.*g*. 107 nm Eu chelate-doped polystyrene nanoparticles in time-resolved fluorescence microscope)^12^ or for counting particles from microscopic droplets fitting the single field of view of the microscope (*e*.*g*. gold nanoparticles in piezo-dispensed microdroplets in scanning electron microscope).^13^ On the other hand, theoretical limits, and uncertainty sources were not investigated. The EVA was also overlooked in reviews dedicated to the methods of nanoparticle quantification.^1,2^ The reasons for this limited use can be (1) contradicting requirements for sensitivity and the field of view (tends to image either large particles or working with impractically small volumes/fields of view),^7^ (2) the need for counting large numbers of nanoparticles (counting uncertainty decreases with number of counted nanoparticles), (3) nanoparticle spatial organization during the droplet drying (causing nanoparticle overlaps and subsequent miscounting).^14^ We solved these problems by (1) stitching multiple micrographs of small fields of view ultimately covering macroscopic scales, (2) artificial intelligence automated the counting of nanoparticles, and (3) nanoparticle clustering was prevented by introducing ultralow gelling agarose. As a model, an aqueous dispersion of ∼60 nm Tm^3+^-doped photon-upconversion nanoparticles (UCNPs) coated in carboxylated silica shells (UCNP-COOHs) are quantified. Uncertainty sources are discussed and theoretical limits are analyzed. A wider applicability is tested by imaging ∼80 nm Nile red-doped polystyrene nanoparticles (NileNPs) and ∼90 nm silver nanoparticles (AgNPs). See the Supporting Information (SI) for nanoparticle synthesis (SI Note S1) and characterization (SI Note S2).

**Scheme 1:**
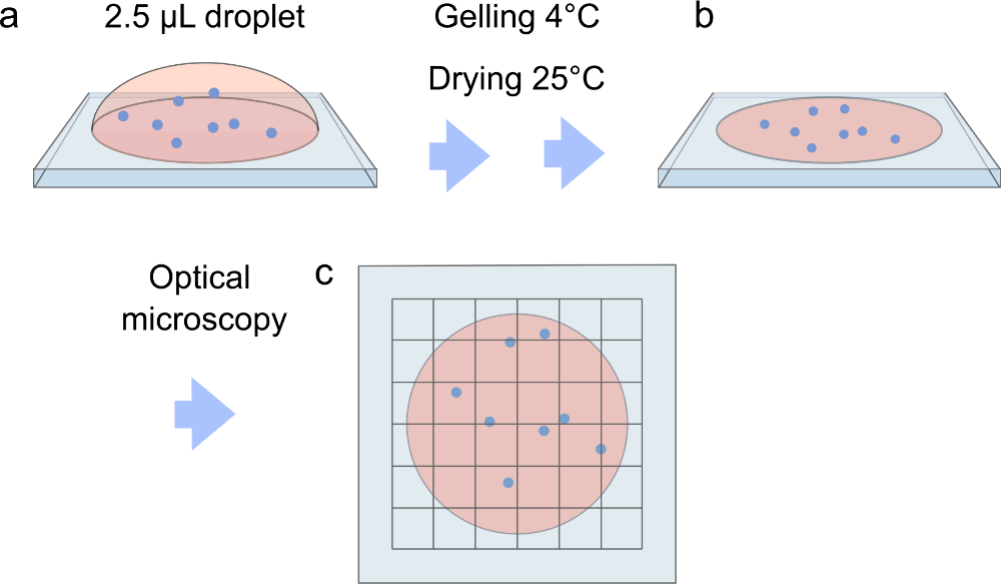
EVA (a) Nanoparticles (blue dots) in ultra-low-gelling agarose are dispensed on a glass substrate. (b) After gelling and drying, (c) the entire droplet is covered with a grit of 6 × 6 images, the images are stitched, and nanoparticles are counted.

TEM showed ∼60 nm oleic acid-capped NaYF_4_ UCNPs doped with 18% Yb^3+^ and 2% Tm^3+^ (Figure S1, SI Note S2). The UCNP-COOHs were formed by coating the UCNPs with a ∼ 5 nm thick carboxylated silica^15^ (Figure 1a, Figure S2, SI Note S2) and were stored as a concentrated aqueous dispersion (11.8 ± 0.24 mg mL^–1^). The sizes were confirmed by dynamic light scattering estimating hydrodynamic diameters at 69 and 78 nm for oleic acid-capped UCNPs and UCNP-COOHs, respectively (Figure 1b). The dispersion of UCNP-COOHs was virtually aggregate-free as confirmed by agarose gel electrophoresis (Figure 1c). Under the excitation of 976 nm, the UCNP-COOH emitted strongly at near-infrared (802 nm, Figure 1d), and in the epiphoton-upconversion microscope appeared as diffraction-limited spots with a full width at half-maximum of 1.8 px (1.2 μm, Figure 1e,f, SI Note S3, Figure S3).

**Figure 1:**
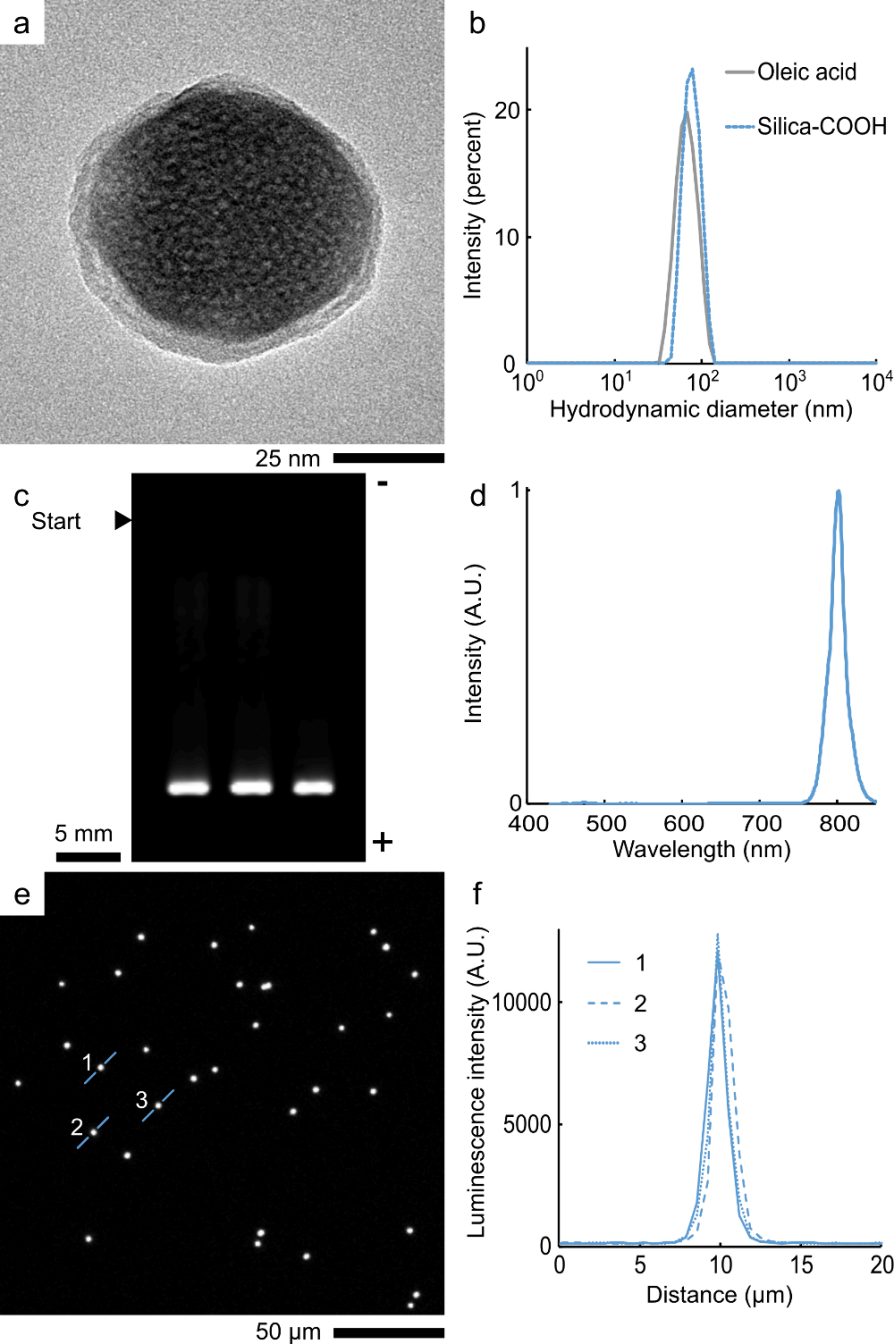
Nanoparticle properties. (a) TEM image of UCNP-COOHs. (b) Hydrodynamic diameters of UCNPs with different surface modifications. (c) UCNP-COOH agarose electrophoresis. (d) The emission spectrum of UCNP-COOHs in water. (e) Epiphoton-upconversion microscopy of UCNP-COOHs in the dried droplet (exposure 5 s). (f) Nanoparticle spot cross sections as indicated in (e). (c-f) Excitation 976 nm. Emission (c) 764–822 nm, and (e,f) 800 ± 25 nm.

For preparing droplets, the stock dispersion of UCNP-COOHs was diluted in ultralow gelling agarose (final concentration 1% w/w, SI Note S3,S4) supplemented with 200 μM NH_4_F protecting the nanoparticles from dissolution.^16^ The gelling temperature of agarose at 8–17 °C allowed work at laboratory temperature. A pipet dispensed the droplets onto a 170 μm thick cover glass (2.5 μL nominal droplet volume, see SI Note S4 for a precise volume calibration). The droplet gelled in the refrigerator (4 °C, 30 min), and then dried at laboratory temperature (25 °C, 60 min). The diameter of droplets was ∼3 mm. With a frame size of 674 μm × 674 μm, the entire droplet was covered with a regular grid of 6 × 6 micrographs in 500 μm steps. The micrographs’ small “pincushion” distortion was calibrated on a rectangular grid and corrected by a Discorpy software library^17^ with subpixel accuracy. The overlaps between adjacent images navigated the stitching algorithm of the FIJI^18^ stitching plugin.^19^ The convolutional neural network of U-net architecture localized the UCNP-COOHs in the micrographs (Figure 2a, Figure S3,S4, SI Note S5).^20^ The localized UCNP-COOHs were counted and subjected to a more detailed analysis when the brightness and the distances between nanoparticles were quantified. The histogram of UCNP intensities revealed one major maximum, which confirmed the sample was virtually free of aggregates (Figure 2b).^11^ The cumulative distribution of distances between the closest-neighboring UCNP-COOHs was well-fitted with a theoretical model^21^ for randomly distributed points (Figure 2c, SI Note 4). The agreement with this model confirms a lack of UCNP-COOH structural organization within the droplet (such as accumulating around the droplet edge).^14^ The number concentration *C* was calculated from eq 1 (*N* is the number of nanoparticles, *V* is the droplet volume, and *D* is the dilution factor):
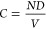
1We found 4438 ± 96 nanoparticles per droplet resulting in the number concentration of (1.75 ± 0.04) × 10^13^ mL^–1^ (mean ± standard deviation from seven droplets, see SI Note S4 for a precise dilution and droplet volume calibration). Standard TEM/gravimetric analysis^1^ and nanoparticle counting in anisotropically collapsed gels^11^ provided reference concentrations. First, UCNP-COOHs were characterized by TEM and the average nanoparticle mass of 596 ag was calculated from its size, shape, and material densities (SI Note S2). The number concentration (1.98 ± 0.04) × 10^13^ mL^–1^ was calculated from the mass concentration of the stock dispersion after dividing by the mass of a single nanoparticle (mean ± standard deviation from three repeated weighing). Alternatively, the number concentration (1.81 ± 0.17) × 10^13^ mL^–1^ was estimated from microlayers of anisotropically collapsing gels (mean ± standard deviation from three repeated gel preparations). The 13% deviation of the TEM/gravimetric approach from EVA is probably caused by the method’s susceptibility to geometrical modeling of nanoparticle shape and size and densities of materials (generally not easily accessible for nanomaterials).^1,11^ The 4% deviation of the concentration estimated in the anisotropically collapsing gel can be explained by the imprecision of agarose microlayer casting.^11^

**Figure 2:**
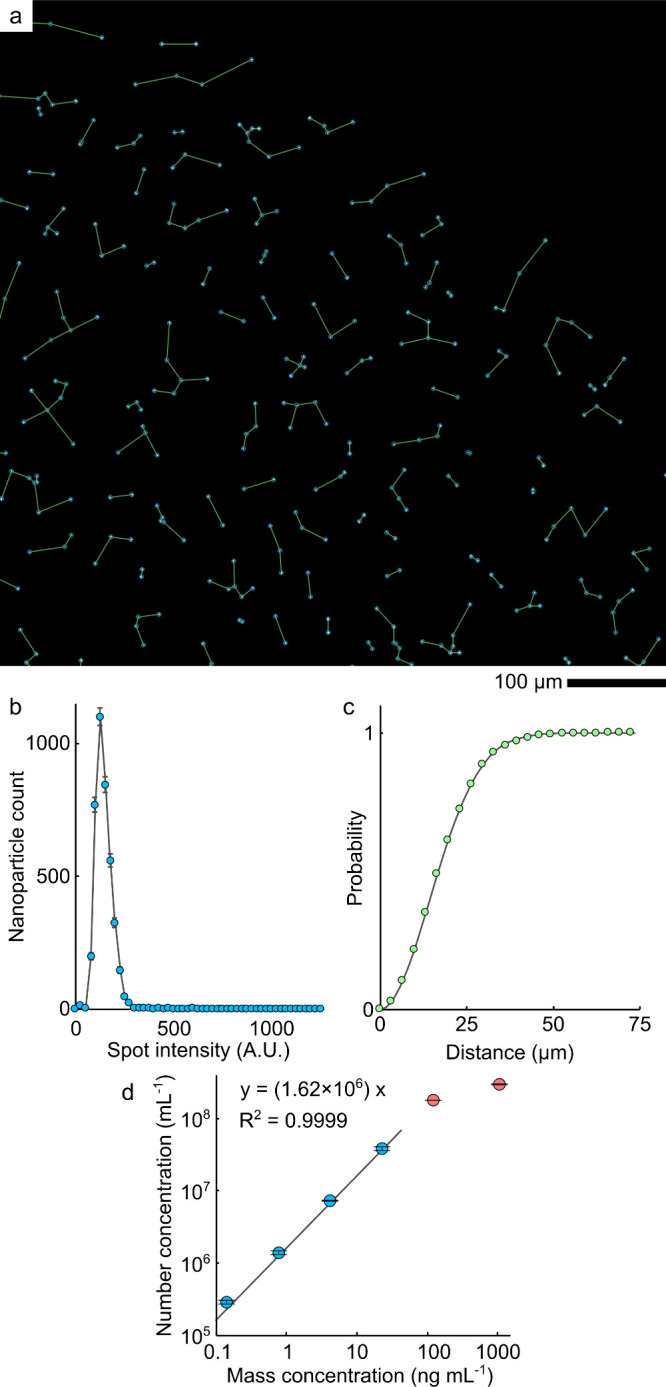
Counting UCNP-COOHs in dried droplets. (a) Annotated micrograph of UCNP-COOHs close to the droplet edge (exposure 5 s, excitation 976 nm, emission 800 ± 25 nm). Blue circles mark nanoparticle localization, and green lines connect the nearest neighbors. (b) The histogram of UCNP-COOH spot intensities (error bars are the square roots of nanoparticle counts). (c) The cumulative distribution of nearest-neighbor distances fitted with the theoretical model (gray line). (d) Dilution series of UCNP-COOHs fitted with a linear function with a zero intercept in a log–log plot (blue circles). Red circles are concentrations out of the linear range. Error bars indicate the standard deviation of two repeats (two droplets).

Unfortunately, the complexity of uncertainty modeling for TEM/gravimetric analysis and anisotropically collapsing agarose gels prevented us from its evaluation. In contrast, the EVA offered straightforward uncertainty modeling as its most significant advantage. According to eq 1, there are only three input quantities—the overall number of nanoparticles in analyzed droplets, dilution of the UCNP-COOH stock dispersion, and overall volume of analyzed droplets (SI Note S4). From these inputs, the number concentration of UCNP-COOH stock dispersion was (1.75 ± 0.05) × 10^13^ mL^–1^ (mean value ± standard uncertainty, relative standard uncertainty 2.7%). The 95% uncertainty coverage interval was from 1.66 × 10^13^ mL^–1^ to 1.84 × 10^13^ mL^–1^. Interestingly, the EVA does not involve precise calibration of measured lengths, which is a unique advantage among other microscope techniques.

As an absolute counting assay, the EVA possesses theoretical limits, which are uncommon among relative assays utilizing a calibration curve—the limits are dependent on the size of analyzed volumes. In this context, we defined the theoretical limit of detection as the lowest concentration when at least one nanoparticle in the overall evaporated volume is found with 99% probability. From the Poisson distribution, it corresponds to ∼4.6 nanoparticles in the overall droplet volume (here ∼270 mL^–1^). As a limit of quantification, we defined the concentration when the Poisson noise of nanoparticle counting is 10%, which refers to 100 nanoparticles in the overall droplet volume (here ∼5900 mL^–1^). The uncertainties of nanoparticle counting translate to optimal working range (SI Note S4). A balance should be set between the Poisson noise of counting and nanoparticle overlaps described by the distribution of the nearest-neighbor distances. Assuming the wished relative standard uncertainty of counting at 2.5% (approximately the relative standard uncertainty of overall droplet volume, SI Note S4), the optimal working range was ∼0.1–200 × 10^6^ mL^–1^. In a dilution series (Figure 2d, SI Note S6, Figures S5–S16), the estimated number concentrations were directly proportional to the nanoparticle mass concentrations with the coefficient of determination R^2^ = 0.9999 in a concentration range 0.15–23 ng mL^–1^ (0.24–38 × 10^6^ mL^–1^). We consider the concentration 0.24 × 10^6^ mL^–1^ a practical limit of detection and quantification–lower concentrations resulted in low nanoparticle counts preventing imaging and stitching. At a concentration of 126 ng mL^–1^ (204 × 10^6^ mL^–1^), the droplets already contained ∼430 thousand nanoparticles. For frequent nanoparticle overlaps (Figure S7), we excluded this concentration from the linear range, although the deviation from linearity was small. See Table S3 for a comparison of the limits of other “nanoparticle counting” methods.

Without substantial nanoparticle clustering, EVA was compatible with buffer solutions (45 mM Tris, 45 mM H_3_BO_3_, pH 8.6), biological matrices (10× diluted bovine plasma, and 275× diluted orange nectar), and organic solvents (chloroform with 0.05% (w/v) polystyrene for oleic acid capped UCNPs; SI Note S6, Figures S17–S20). Beyond UCNP-COOHs, fluorescent NileNPs were imaged with epifluorescence microscopy (Figure S21), and bright-field and dark-field microscopy imaged plasmonic AgNPs (Figures S22,S23). Compared to UCNP-COOHs, the signals from these nanoparticles are less specific and the images are noisier complicating nanoparticle counting. However, considering the rapid development of artificial intelligence, this limitation will soon melt away.^20^ A substantial EVA improvement is expected with microscope automation–the capability for thousands of images per droplet will increase imaging sensitivity and decrease counting uncertainty.

In conclusion, image stitching, artificial neural networks, and agarose gel refined the properties of EVA. The number concentration of ∼60 nm UCNPs was estimated with a relative standard uncertainty of 2.7%. Imaging fluorescent NileNPs and plasmonic AgNPs confirmed the wider applicability.

## Supporting Information Available

Nanoparticle synthesis, characterization, optical microscopy, measurement uncertainties, nanoparticle counting, and micrographs (PDF)
