# Supplementary material for: Optical Microscopy and Deep Learning for Absolute Quantification of Nanoparticles on a Macroscopic Scale and Estimating Their Number Concentration
Source: Anal Chem. 2025 Jan 31;97(5):2588–92. doi: 10.1021/acs.analchem.4c05555 (PMC11822731; doi:10.1021/acs.analchem.4c05555)
Supplement: Supplementary file 1 — ac4c05555_si_001.pdf [file ac4c05555_si_001.pdf]

Supporting Information

Optical Microscopy and Deep Learning for Absolute Quantification of Nanoparticles on a Macroscopic Scale and Estimating Their Number Concentration

Antonín Hlaváček,[a]* Kateřina Uhrová,[a] Julie Weisová,[a] Hana Brožková,[a,b] Naděžda Pizúrová[c]

[a]Institute of Analytical Chemistry of the Czech Academy of Sciences, Brno, Czech Republic

[b]Department of Chemistry, Faculty of Science, Masaryk University, Brno, Czech Republic

[c]Institute of Physics of Materials of the Czech Academy of Sciences, Brno, Czech Republic

*E-mail: hlavacek@iach.cz

Table of contents

Note S1. Nanoparticle synthesis....2
Note S2. Nanomaterial characterization....2
Estimating nanoparticle hydrodynamic diameters by dynamic light scattering....2
Transmission electron microscopy....2
Gel electrophoresis....2
Estimating the mass concentration of photon-upconversion nanoparticles....2
Estimating the nanoparticle mass from transmission electron microscopy images....2
Estimating number concentration from anisotropically collapsing gels....3
Note S3. Optical microscopy....3
Sample preparation....3
Microscope setup....3
Epiphoton-upconversion and epifluorescence microscopy....3
Bright-field microscopy....4
Dark-field microscopy....4
Scanning the entire droplet and stitching the microscope images....4
Note S4. Measurement uncertainties....4
Evaluating combined uncertainties....4
Uncertainty of nanoparticle counting....4
Uncertainty of weighing....5
Densities of used liquids....5
Uncertainty of dilution factor....5
Uncertainty of droplet volumes....6
Note S5. Nanoparticle localization and counting....7
Note S6. Additional experiments....8
UCNP-COOH dilution series....8
Buffer solution....8
Bovine plasma....8
Orange nectar....8
Chloroform....8
Note S7 Method comparison....9
Transmission electron microscopy....10
Optical microscopy....12
References....24

Note S1. Nanoparticle synthesis

Photon-upconversion nanoparticles[1], fluorescent Nile red-doped polystyrene nanoparticles[2], and plasmonic silver nanoparticles[3] were prepared as reported previously.

Note S2. Nanomaterial characterization

Estimating nanoparticle hydrodynamic diameters by dynamic light scattering

Dynamic light scattering was used for measuring the nanoparticle hydrodynamic diameters. The diluted sample (~0.1 mg mL^{-1}) was dispensed into a glass cuvette with a 10 mm optical path and inserted into the Zetasizer Nano ZS (Malvern Panalytical). Data were collected with a standard measurement setting.

Transmission electron microscopy

To prepare samples for transmission electron microscopy (TEM), the oleic acid-capped nanoparticles were diluted to 0.1 mg mL^{-1} in cyclohexane, and UCNP-COOHs were diluted in water to 0.1 mg mL^{-1}. The samples were dropped on the TEM grids (holey carbon coated Cu grid), and the excess liquid was absorbed by a paper tissue and dried freely on air. The micrographs were taken from a dry grid (Talos F200i, Thermo Fisher Scientific).

Gel electrophoresis

The dispersion of Tm^{3+} UCNP-COOHs (0.9 µL, 11.8 mg mL^{-1}) was mixed with 89.1 µL TB buffer (50 mM tris(hydroxymethyl)aminomethane, 50 mM H_3BO_3, pH 8.6) and glycerol (10 µL, 90% w/w glycerol/water mixture). The resulting sample was loaded on 0.75% agarose gel (Agarose NEEO ultra-quality ROTIGarose low electroendosmosis, Carl Roth) in three replicates (18 µL per well). The TB buffer served also as an electrophoresis buffer. The electrophoresis ran at a constant voltage of 150 V for 30 min (Owl C2-S Micro Electrophoresis System, Thermo Fisher Scientific). The developed gel was scanned for photon-upconversion emission at wavelengths 764-822 nm (976 nm excitation) with a resolution of 200 µm by utilizing a laboratory-built laser scanner.

Estimating the mass concentration of photon-upconversion nanoparticles

As described previously,[1] an empty 4 mL glass vial was precisely weighted (analytical balance PX225DM, OHAUS Europe). A volume of 250 µL of nanoparticle stock dispersion was dispensed into the vial. The vial was placed on a heater in a fume hood, and the water was evaporated. Then the vial was put into a laboratory oven and kept there for 90 min at 550 °C to remove the remaining water and organic compounds. After cooling to room temperature, the vial was precisely weighted. The mass concentration of nanoparticles was estimated from the mass differences of the glass vials and the applied volume of nanoparticle dispersion.

Estimating the nanoparticle mass from transmission electron microscopy images

To estimate the mass of a single nanoparticle from transmission electron microscopy (TEM), the volume of the lanthanide-doped $NaY_{0.80}Yb_{0.18}Tm_{0.02}F_4$ core was estimated separately from the volume of the silica shell. These volumes were multiplied by the densities of respective materials to provide the mass of a single average nanoparticle. The density of non-doped $NaYF_4$ and silica are 4130 kg m^{-3},[4] and 1900 kg m^{-3},[5] respectively. The density of the doped $NaYF_4$ was increased proportionally to the content of doped lanthanides and their atomic masses.[6] Thus the calculated density of $NaY_{0.80}Yb_{0.18}Tm_{0.02}F_4$ core was 4497 kg m^{-3}. The nanoparticles were modeled as a spheroid (Scheme S1, Figure S1,S2):

L_1 length of the spheroid major axis 63.1 ± 1.9 nm

L_2 length of the spheroid minor axis 56.9 ± 2.5 nm

T thickness of the silica shell 4.7 ± 0.8 nm

D_{core} the density of core 4497 kg m^{-3}

D_{silica} the density of silica 1900 kg m^{-3}

V_{core} the volume of the core

V_{silica} the volume of the silica shell

m mass of a single nanoparticle

$V_{core} = 4 / 3 \times \pi \times (L_1/2) \times (L_2/2)^2$

$V_{silica} = 4 / 3 \times \pi \times (L_1/2 + T) \times (L_2/2 + T)^2 - V_{core}$

Calculation of the nanoparticle mass:

$m = (V_{core} \times D_{core} + V_{silica} \times D_{silica})$

m = 596 ag

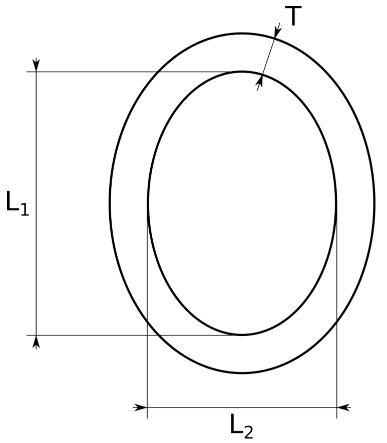

Scheme S1. The model used for calculating the masses of a single UCNP-COOH.

Estimating number concentration from anisotropically collapsing gels

An absolute counting method in anisotropically collapsed agarose gel was used for estimating the number concentrations of UCNP-COOHs as described previously.[7] The nanoparticles were immobilized in a submicron agarose layer of a known thickness (38 ± 1.2 µm), imaged as bright spots by an epiphoton-upconversion microscope (excitation 976 nm, emission 800 ± 25 nm), and counted. The number concentration of nanoparticles was estimated from the thickness of the agarose gel before drying, the area of dried gel where the nanoparticles were counted, and the number of counted nanoparticles.

Note S3. Optical microscopy

Sample preparation

At laboratory temperature, the sample of nanoparticles was mixed with aqueous dispersion of 1.1% (w/w) ultra-low gelling agarose (gelling temperature 8-17°C, FMC BioProducts), and droplets of this mixture were dispensed with a hand-held pipette on a cover glass (thickness 170 µL). After droplet dispensing, the glass slides were closed in a Petri dish and placed into the refrigerator to gel the agarose (30 min, 4°C). In the next step, the Petri dish was opened and the droplets dried at laboratory temperature. After drying, the cover glass slides were attached to the microscope glass slides (thickness 1 mm). For epifluorescence and epiphoton-upconversion microscopy, the cover glasses were attached by a double sided adhesive tape (thickness ~80 µm), and the dried droplets became enclosed between the glass slides. For bright-filed microscopy, the cover glass was attached by a transparent mounting medium (ROTI Histokitt, Carl Roth) without having any air between the glass slides.

Microscope setup

A laboratory-built microscope with epiphoton-upconversion, epifluorescence, bright-field, and dark-field imaging modalities was used with air immersion objective (magnification 20×, numerical aperture 0.50, Nikon, single frame size 674 µm × 674 µm). Electron multiplying charge-coupled device camera iXon Life 888 (Oxford Instruments) with a 1024 px × 1024 px sensor was utilized for image recording. The image of the sample was projected on the camera sensor by a tube lens of 200 mm focal length (Thorlabs). The imaging modalities were selected depending on the optical properties of observed nanoparticles.

Epiphoton-upconversion and epifluorescence microscopy

Near-infrared excitation laser was used for imaging the photon-upconversion nanoparticles (wavelength 976 nm, Roithner Lasertechnik). The power illuminating the sample was 1.9 W and the sample illumination intensity was ~400 W cm^{-2}. A laser of a shorter wavelength was used for fluorescent nanoparticles (wavelength 520 nm, Thorlabs). The power illuminating the sample was 76 mW and the sample illumination intensity was ~17 W cm^{-2}). The laser beam was introduced into the microscope by passing through a rotating diffuser (Thorlabs). The unwanted wavelengths in the laser beam were removed by input optical filter (950 nm long-pass filter for photon-upconversion, 520 ± 20 nm band-

pass filter for fluorescence, Thorlabs). The laser beam was reflected by a dichroic mirror into the microscope objective (900 nm short-pass mirror for photon-upconversion nanoparticles, and 550 nm long-pass mirror for fluorescent nanoparticles, Thorlabs). The wavelengths of the luminescence emission were selected by an output filters (800 ± 25 nm band-pass filter for Tm^{3+} doped photon-upconversion nanoparticles, and 650 ± 25 nm band-pass filter for Nile red-doped polystyrene nanoparticles, Edmund Optics). The camera was carefully calibrated to compensate for the irregularities of sample illumination, dark signal, and the uneven sensitivity of camera pixels.

Bright-field microscopy

A white light-emitting diode was used for bright-field microscopy with a laboratory-built condenser illuminating the sample. A 475 ± 25 nm band-pass filter was inserted behind the microscope objective to block other wavelengths from reaching the camera. Advanced image recording and processing were used to improve the signal-to-noise and signal-to-background ratios. Three types of image stacks (100 raw images per stack) were recorded to construct the final image. With an exposure time of 30 ms, the stack of 100 raw images was recorded in 16 s. (A) stack of raw sample images (A stack, all raw images recorded in the same area of the sample), (B) reference stack (B stack, the microscope stage was randomly moving over the sample during the stack recording), (C) dark signal stack (C stack, signal with the light source switched off). The median images were calculated through each stack A, B, and C resulting in images mA, mB, and mC, respectively. The final image fA of the sample was then constructed by subtracting and dividing between median images: fA = (mA - mC) / (mB - mC). Thus, the final image is the relative transmission of the light. The nanoparticles appeared as dark spots as they caused the attenuation of the transmitted light.

Dark-field microscopy

A white light-emitting diode was used for dark-field microscopy with a laboratory-built condenser illuminating the sample. A 475 ± 25 nm band-pass filter was inserted behind the microscope objective to block other wavelengths from reaching the camera.

Scanning the entire droplet and stitching the microscope images

Motorized microscope stage moved the droplet around the microscope objective. The stage was controlled by an Arduino board from a Java graphical interface. For microscopy of the entire droplet surface, the stage with the sample moved in a two-dimensional grid with a step of 500 µm (6 × 6 micrographs). The geometrical distortion of images was calibrated on a rectangular grid distortion target with horizontal and vertical lines spaced by 50 µm in a bright-field mode with 800 ± 25 nm band-pass filter. The distortion was then corrected by the Discorpy software library.[8] The corrected images were stitched in FIJI image processing software[9] by a standard stitching plugin[10] to form a single image covering the entire droplet surface.

Note S4. Measurement uncertainties

Evaluating combined uncertainties

To evaluate combined uncertainties of measurements, the modeling approach according to "Evaluation of measurement data — Supplement 1 to the Guide to the expression of uncertainty in measurement — Propagation of distributions using a Monte Carlo method".[11] The uncertainties of input quantities were described by Gaussian, rectangular, or gamma distributions of suitable parameters, and the uncertainty was propagated by Monte Carlo simulation with 5×10^6 random sampling runs.

Uncertainty of nanoparticle counting

Poisson noise. Poisson noise is caused by sampling nanoparticles randomly from a dispersion, and its uncertainty is simulated by sampling from a gamma distribution. In seven droplets, 31063 nanoparticles was counted, the standard uncertainty was 176 nanoparticles, and the relative standard uncertainty was 0.6% (RSU, *i.e.*, standard uncertainty divided by a mean value).

The correctness of nanoparticle localization. Because ground true data are not easily available for single-molecule or single-nanoparticle localization, the network performance was evaluated by a human expert, similar to previous reports.[12] The nanoparticle localization in three droplets was analyzed. The network localized 13166 nanoparticles. The human expert then recognized 31 (0.24%) false-positive and 4 (0.03%) false-negative localizations. From this, the uncertainty of counting can be assigned with rectangular distribution from -0.24% to +0.24% nanoparticle counting. However, to avoid uncertainty underestimating, we assigned a wider rectangular distribution from -0.5% to +0.5% of nanoparticles counted. Then, the counting RSU of 0.29% can be received after dividing 0.5% by √3.

Spot overlaps. A peculiar aspect of nanoparticle counting is the overlap between diffraction-limited spots of two nanoparticles, which are closer than the spatial resolution of the microscope. As a resolution criterion, we considered the full width at half maximum of the diffraction-limited spots, which was estimated at 1.8 px (1.2 µm, measured by ImageJ plugin GDSC SMLM).[9,13] The localizations were made with single-pixel precision, therefore, the length of the diagonal in a rectangle of 2 px × 2 px should be considered an efficient resolution. The diagonal length is 2.8 px and this was further rounded to 3 px (2.0 µm) for a convenience and to avoid underestimating the uncertainty of overlaps. The percentage of nanoparticles closer than the efficient microscope resolution was then calculated from the nearest neighbor distance distribution of randomly distributed points.[14] Its cumulative distribution function is:

$$P(r) = 1 - e^{(-\lambda \pi r^2)}$$

where *P(r)* is the probability that the distance between nanoparticles is smaller than the distance r, λ is the number of nanoparticles per image area, e is Euler's number, and π is the number pi. The probability that the distance between nanoparticles is smaller than the resolution is calculated by setting r = efficient resolution = 3 px (2.0 µm). The nanoparticle density was 0.00034 per px^2 (0.00015 per µm^2). As a result, 1% of nanoparticles was closer than the efficient resolution limit. These nanoparticles can introduce a negative bias into the nanoparticle counting (fewer nanoparticles are counted than actually present). On the other hand, we are not sure if these nanoparticles are present. Additionally, several nanoparticles per droplet containing on average 4438 nanoparticles were found with nearest neighbor distances of 3 px or smaller. Therefore, rather than bias, we simulated the spot overlap uncertainty as a rectangular distribution from -1% to 1% of nanoparticle count (translates to the RSU of 0.6% after dividing by √3).

Uncertainty of weighing

Analytical balance PX225DM (OHAUS Europe) was used within all experiments. The balance was regularly adjusted using the internal adjustment. The weighing uncertainties were quantified by evaluating balance readability (rounding of the digital reading) and measuring repeatability and calibration uncertainty by the OIML E2 test weights (KERN & SOHN, eleven standards in the range from 1 mg to 100 g). Air buoyancy correction was applied for weighed aqueous dispersions.

Densities of used liquids

The densities of UCNP-COOH stock dispersion, 200 µM aqueous NH_4F, 1.0% (w/w) ultra-low gelling agarose supplemented with 200 µM NH_4F, and 1.1% (w/w) ultra-low gelling agarose supplemented with 200 µM NH_4F were measured by Gay-Lussac pycnometer at laboratory temperature (25°C). The estimated densities were 1005.9 ± 0.18 mg mL^{-1}, 997.07 ± 0.05 mg mL^{-1}, 1000.39 ± 0.05 mg mL^{-1}, and 1000.65 ± 0.05 mg mL^{-1}, respectively (mean ± standard uncertainty). As a control, the density of water was measured at 997.07 ± 0.05 mg mL^{-1} (mean ± standard uncertainty), which compares well with the tabular value of 997.048 mg mL^{-1} for water at 25°C.[15]

Uncertainty of dilution factor

Scheme S2 presents the dilution of UCNP-COOH stock dispersion. The UCNP-COOHs were diluted in two steps in 200 µM aqueous NH_4F when fluoride ions protected the UCNP nanocrystals from dissolving.[16] The sample was further diluted in 1.1% (w/w) ultra-low gelling agarose supplemented with 200 µM NH_4F. The gelling temperature of agarose was 8-17°C allowing work at laboratory temperature. According to Scheme S2, the volumes V1, V3, and V5 were measured by pipettes. After performing the dilution, the pipettes were calibrated by pipetting volumes in 50 repeats.[17] The calibration provided the mean value of measured volume and standard uncertainty (combined uncertainty of calibration uncertainty, and repeatability, Table S1). For evaluating the uncertainty of the volumetric flask (volumes V2 and V4 in Scheme S2), the combined uncertainty was calculated from calibration uncertainty, filling repeatability, and temperature fluctuations of the laboratory temperature from 24 to 26°C. The results are listed in Table S1. The agarose dispersion was quite viscous and therefore the volume V6 was measured by weighing the agarose dispersion and dividing by its density (Table S1). Finally, the overall dilution was calculated at 9557462 ± 61683 (mean ± standard uncertainty) from calibrated volumes V1-V6 and propagating their uncertainties. The RSU of dilution was 0.7%.

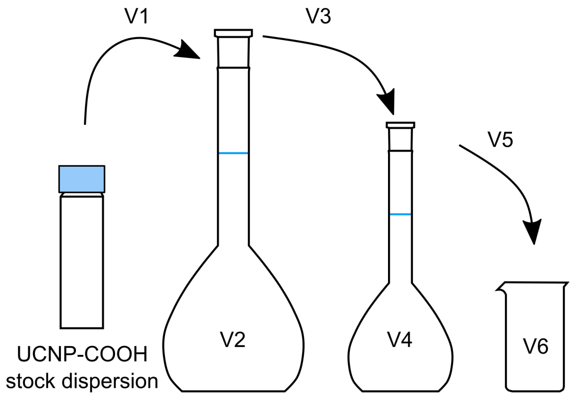

Scheme S2. Dilution scheme of the stock UCNP-COOHs dispersion.

Table S1. Volume calibration.

Volumes V1-V6 – nominal values	Volumes V1-V6 – calibrated values (mean value ± standard uncertainty)
V1; pipette: 140 µL	142.2 ± 0.78 µL
V2; volumetric flask: 500 mL	500 ± 0.16 mL
V3; pipette: 1000 µL	1006.5 ± 2.5 µL
V4; volumetric flask: 250 mL	250 ± 0.10 mL
V5; pipette: 1000 µL	1006.5 ± 2.5 µL
V6; weighing: 10 mL	10007.4 ± 2.8 µL

Uncertainty of droplet volumes

For counting nanoparticles, the droplets (2.5 µL nominal volume) of the sample diluted in agarose were dispensed on a glass substrate. The pipette was calibrated after dispensing the droplets in 50 repeats.[17] The calibration provided the mean volume, calibration uncertainty, and repeatability. These values were used for calculating the uncertainty of the overall volume of all seven analyzed droplets. The RSU of the overall droplet volume was 2.4%, see Table S2 for calibration results.

Table S2. Droplet volume calibration.

Nominal values	Calibrated volume (mean value ± standard uncertainty)
One droplet: 2.5 µL	2.43 ± 0.11 µL
Overall volume of seven droplets: 17.5 µL	17.01 ± 0.40 µL

Note S5. Nanoparticle localization and counting

FIJI-ImageJ was used for visualizing and manual image evaluation.[9] A laboratory-developed convolutional neural network was used for automatic image processing (implemented in the Python programming language using the Keras deep learning interface). The images were recorded in 16-bit pixel depth providing intensity values from ~500 to 65535. Before neural network analysis, the images were logarithmized with the base of two and divided by 16 resulting in values from ~0.56 to 1.0. The localization of spots was performed by a convolutional neural network with a U-net architecture (Scheme S3). The U-net was selected for its already proven capability for localizing diffraction-limited spots.[12,18] Following the previous reports, the model was trained on simulated data.[12] The reasons for using simulated data are two: (A) "Ground-truth" data are not easily available for single particle localization. (B) It is possible to simulate realistic images as the physics of imaging single molecules/nanoparticles is well understood.[12,18] The spots were simulated as two-dimensional Gaussian peaks with a Poisson noise. To introduce the Poisson noise, the simulated intensity was replaced with a random sample from a Poisson distribution having the mean value equivalent to that simulated intensity. The simulated peaks were superimposed on real images to introduce realistic camera noise and background. A mask indicating the positions of simulated nanoparticles was generated for each image. The size of simulated images was 128 px × 128 px. The number of 1 to 50 spots was simulated per image, and the training set contained 6400 image-mask pairs with a 20% validation split. The trained network was tested manually by evaluating real images, see the paragraph "The correctness of nanoparticle localization" in Supporting Note S4 for more details. Once trained, the U-net processed the logarithmized and scaled images and returned maps of spot localization, which were converted to binary masks by thresholding. The binary masks of spot localization were used for counting nanoparticles and measuring the closest distances between nanoparticles by a Python script. To ease manual inspection, circles were drawn around localized nanoparticles, and a line was drawn connecting the nearest neighbors (Figure S3,S4). To restrict the nanoparticle counting to any region of interest (such as the droplet area), an additional mask was created covering the region of interest – nanoparticles out of the mask were not involved in the counting and spot analysis. This allowed for removing artifacts introduced by the stitching algorithm far from the nanoparticle spots (i.e., far from the droplet), whose presence otherwise navigates the stitching algorithm. In these areas, the stitching algorithm introduces rectangles filled with zeros, which cause false positive localization of nanoparticles.

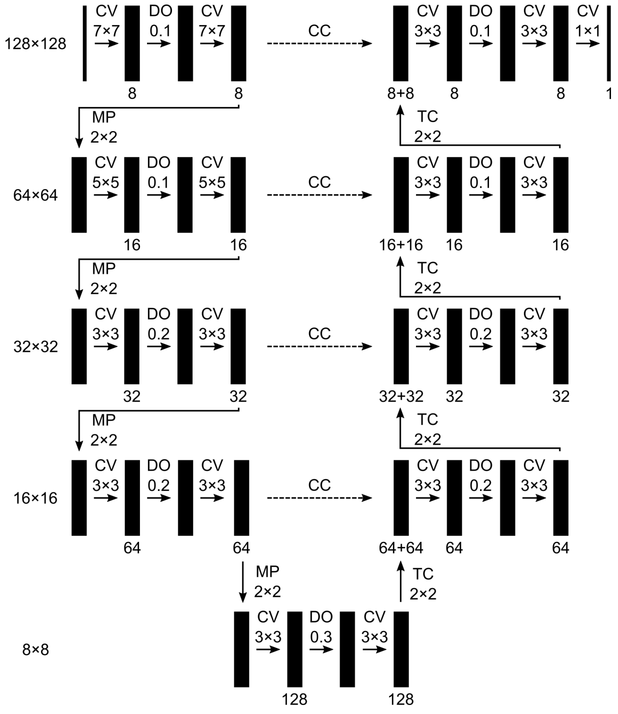

Scheme S3. The scheme of U-net. The U-net is composed of convolution (CV), drop out (DO), max pooling (MP), transposed convolution (TC), and concatenation (CC) layers. In the first part, the network successively reduces the size of the input image (128 px × 128 px) to a set of 128 feature maps of reduced size (8 px × 8 px). In the second part, the output image is built as a map of spot localization (128 px × 128 px).

Note S6. Additional experiments

UCNP-COOH dilution series

The stock dispersion of UCNP-COOHs was gradually diluted in 1% ultra low gelling agarose supplemented with NH_4F to the concentration of 200 µM. The volumes in dilution series preparation were dispensed by pipette and weighed. The weighing and known densities of liquids were used to calculate the dilution in all steps precisely. Droplets (2.46 ± 0.16 µL) of different UCNP-COOHs dilutions were prepared for microscopy as described in Note S3. The mass concentrations in dilution series were 1080, 126, 23, 4.3, 0.79, 0.15, and 0 ng mL^{-1}. Two droplets were analyzed for each dilution. No nanoparticles were observed in the blank experiment, for which no images were recorded (concentration of UCNP-COOHs 0 ng mL^{-1}, *i.e.*, no nanoparticles were added).

Buffer solution

The UCNP-COOHs were diluted to approximately ~3 ng mL^{-1} in 1.1% ultra low gelling agarose supplemented with NH_4F to the concentration of 200 µM. The volume of 1000 µL of this dispersion was mixed with the volume of 100 µL of buffer solution (500 mM tris(hydroxymethyl)aminomethane (Tris), and 500 mM H_3BO_3). The resulting Tris and H_3BO_3 concentrations were 45 mM. Droplets (~2.5 µL) were prepared for microscopy as described in Note S3.

Bovine plasma

The UCNP-COOHs were diluted to approximately ~3 ng mL^{-1} in 1.1% ultra low gelling agarose supplemented with NH_4F to the concentration of 200 µM. The volume of 1000 µL of this dispersion was mixed with the volume of 100 µL of bovine plasma (Sigma-Aldrich), which was previously filtered by a syringe filter with a pore size of 220 nm. Droplets (~2.5 µL) were prepared for microscopy as described in Note S3.

Orange nectar

The UCNP-COOHs were diluted to approximately ~3 ng mL^{-1} in 1.1% ultra low gelling agarose supplemented with NH_4F to the concentration of 200 µM. The volume of 1000 µL of this dispersion was mixed with 100 µL of a diluted orange nectar (prepared by mixing 960 µL of water and 40 µL of the orange nectar; LINEA-NIVNICE), which was previously filtered by a syringe filter with a pore size of 220 nm. Droplets (~2.5 µL) were prepared for microscopy as described in Note S3.

Chloroform

UCNPs capped with oleic acid were diluted to the concentration of approximately ~15 ng mL^{-1} in chloroform supplemented with polystyrene (320 kg mol^{-1}; Carl Roth) to the concentration of 0.05% (w/v). Droplets (~1 µL) on a glass substrate (thickness 170 µm) dried rapidly within a few tens of seconds. The dried droplets were prepared for microscopy as described in Note S3.

Note S7 Method comparison

Table S3. Comparison of single nanoparticle counting methods.

Method	Limit of detection	Linear range	Nanoparticle type	Reference
Evaporation volume analysis	2.4×10^5 mL^{-1}	2.4×10^5–38×10^6 mL^{-1}	60 nm photon-upconversion nanoparticles doped with Tm^{3+}	This work.
Single particle inductively coupled plasma mass spectrometry	1 pg mL^{-1}<br>(5×10^2 mL^{-1})	10–1000 pg mL^{-1}<br>(5×10^3–5×10^5 mL^{-1})	60 nm gold nanoparticles	19
	5 pg mL^{-1}<br>(4×10^3 mL^{-1})	5–500 pg mL^{-1}<br>(4×10^3–4×10^5 mL^{-1})	60 nm silver nanoparticles	
Fluorescence detection in a flow channel	-	6×10^7–6×10^9 mL^{-1}	Quantum dots	20
Light scattering detection in a flow channel	-	2.8×10^6–7.0×10^8 mL^{-1}	43 nm gold nanoparticles	21
Resistive pulse sensing	-	10^7–10^{11} mL^{-1}	160 nm polystyrene nanoparticles	22
Nanoparticle tracking analysis	Particles detected at 10^6 mL^{-1} with a significant deviation from linearity.	10^8–10^9 mL^{-1}	100 nm polystyrene and 100 nm SiO_2 nanoparticles	23
Anisotropically collapsed gel	2×10^6 mL^{-1}	4.4×10^7 to 2.2×10^{10} mL^{-1}	40 nm photon-upconversion nanoparticles doped with Er^{3+}	7
Nanopipet	-	5×10^{10}–5×10^{13}	40 nm Gold nanoparticles	24

Transmission electron microscopy

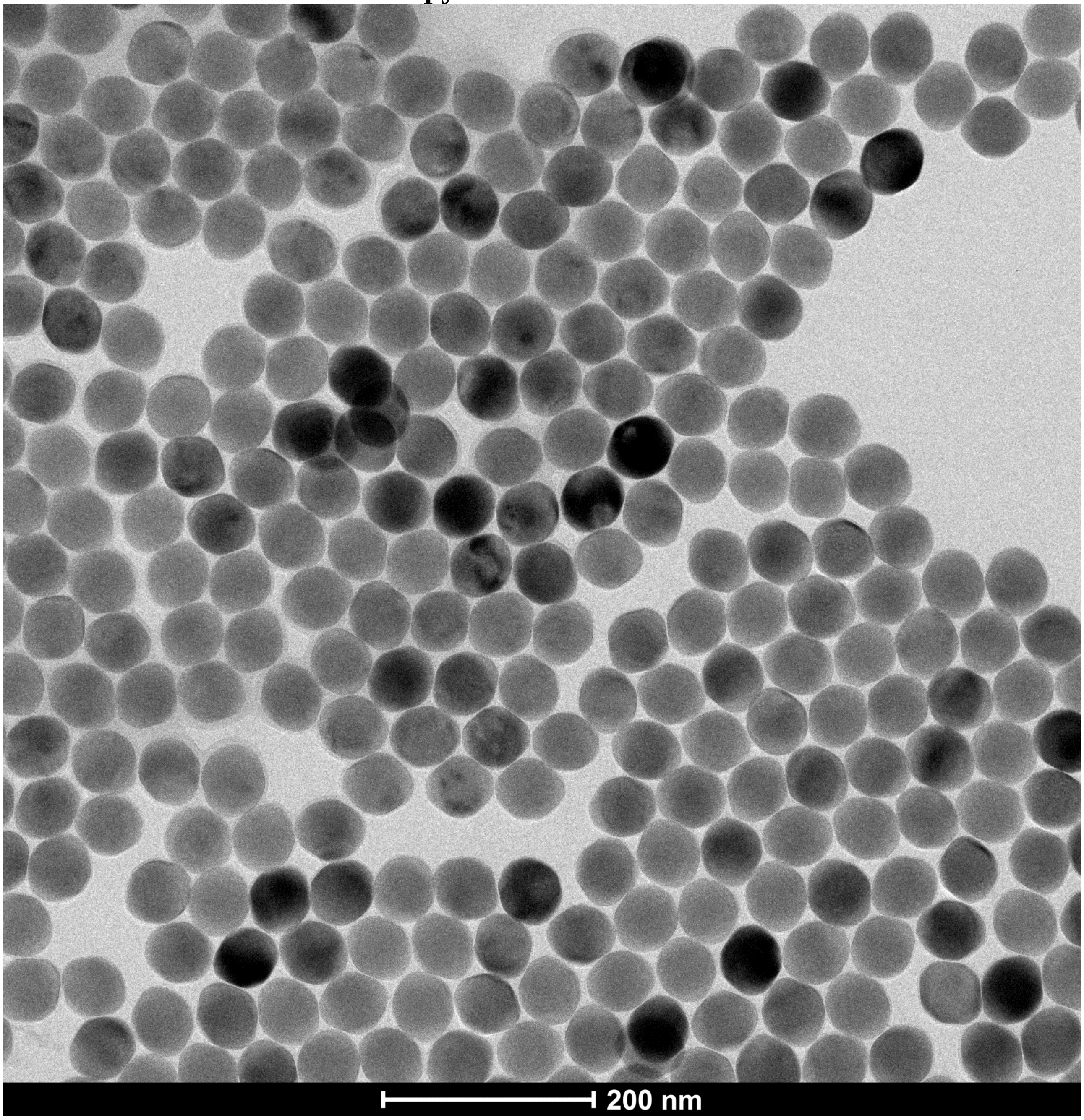

Figure S1. Transmission electron microscopy of the oleic acid-capped UCNPs.

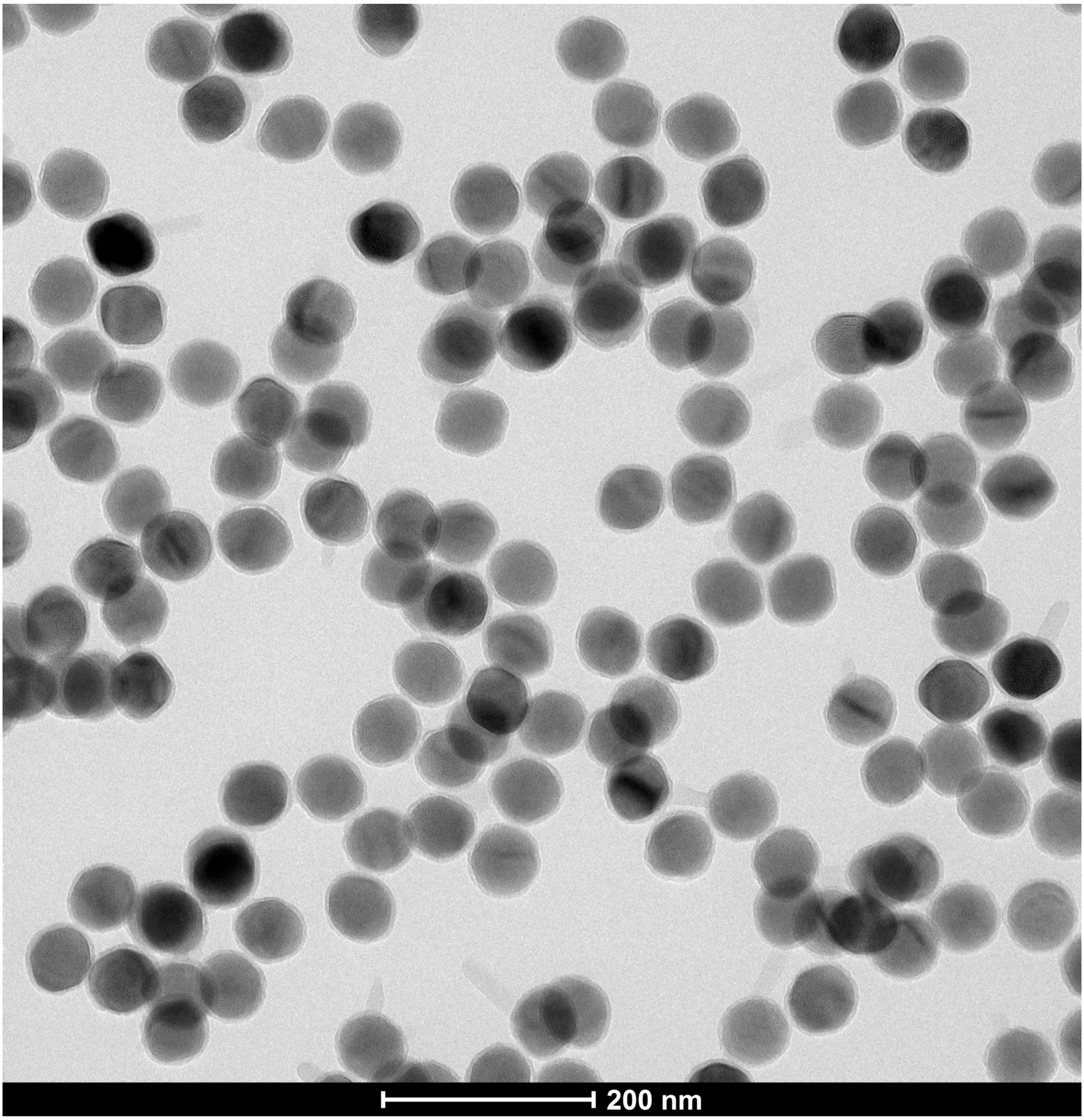

Figure S2. Transmission electron microscopy of the UCNP-COOHs.

Optical microscopy

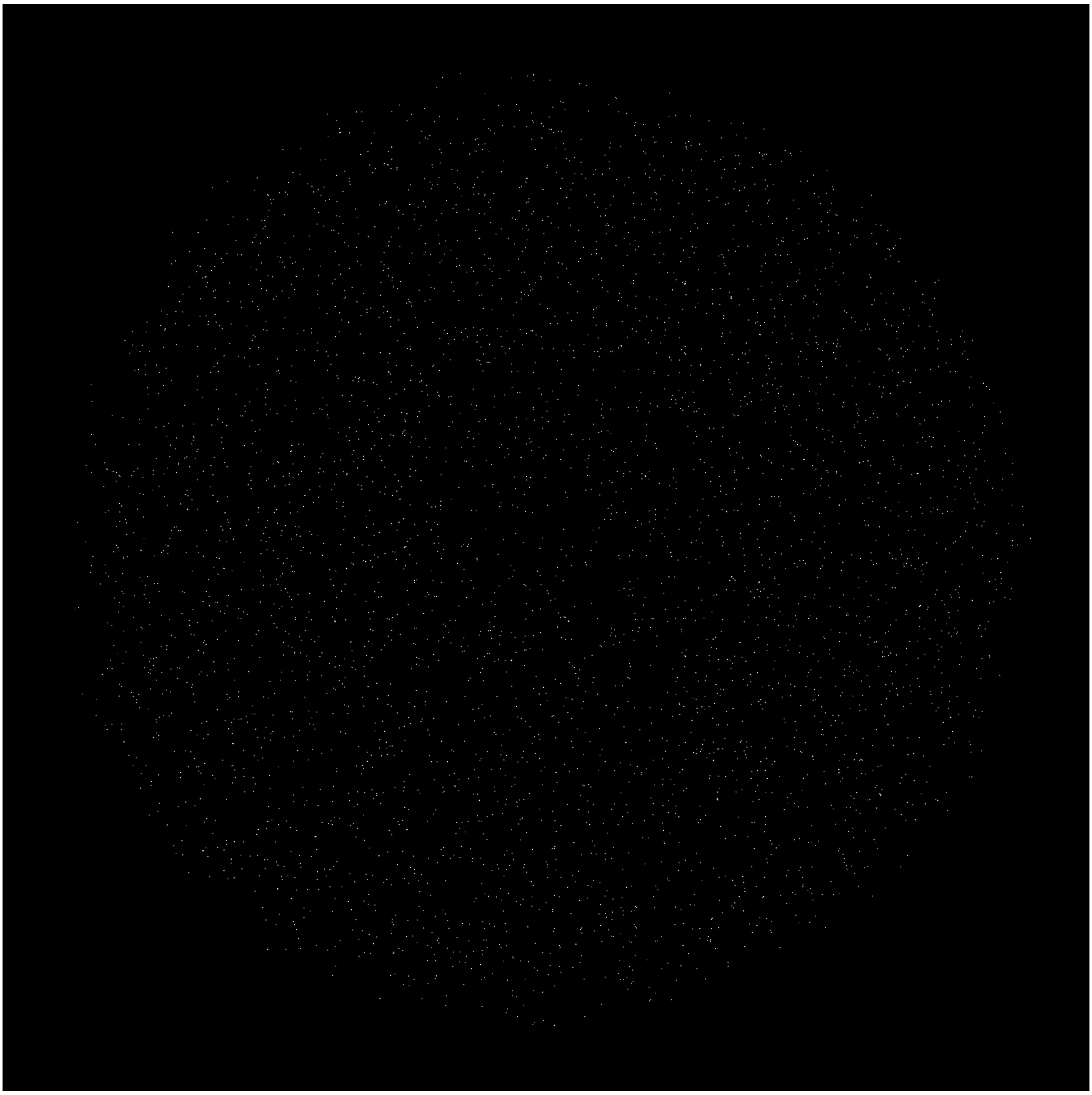

Figure S3. Epiphoton-upconversion microscopy. Epiphoton-upconversion microscopy of UCNP-COOHs in a dried droplet, dispersion volume of 2.5 µL, excitation wavelength 976 nm, emission wavelength 800 ± 25 nm, UCNP-COOHs appear as white spots. Image size 3000 µm × 3000 µm.

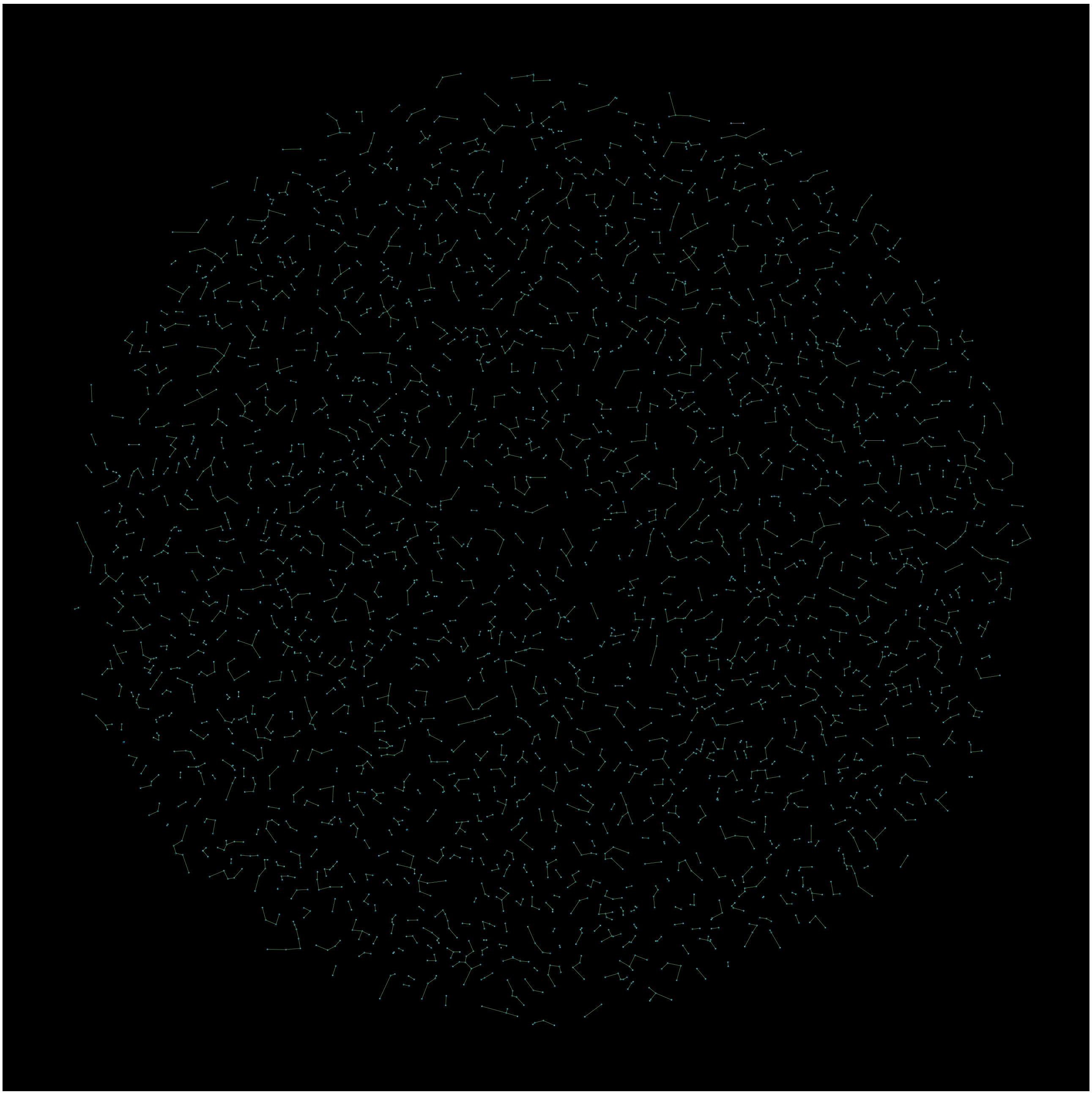

Figure S4. Annotated Figure S3. Blue circles are the localization of UCNP-COOHs. Green lines connect the nearest neighboring UCNP-COOHs. The number of localized UCNP-COOHs is 4226 in this droplet. Image size 3000 μm × 3000 μm.

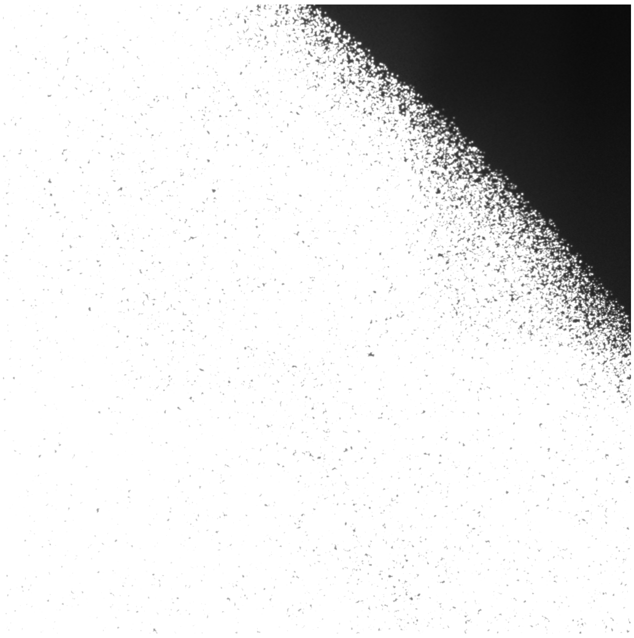

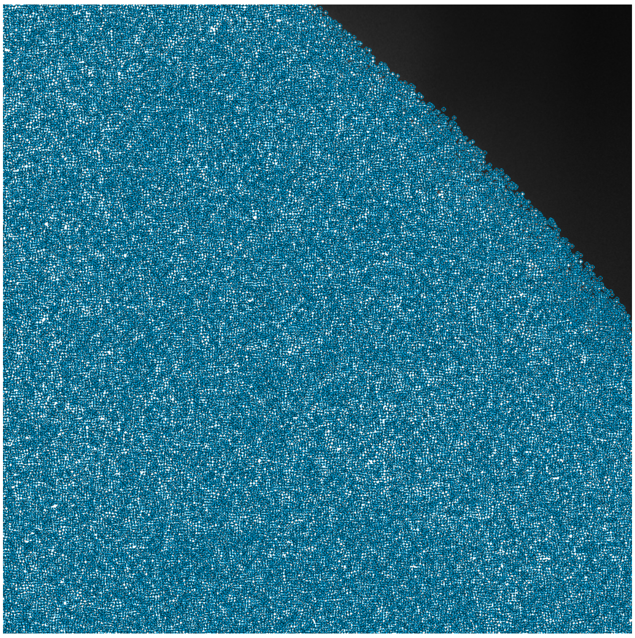

Figure S5. Epiphoton-upconversion microscopy. A part of a dried droplet close to the droplet edge, UCNP-COOH mass concentration 1080 ng mL^{-1}, dispersion volume 2.46 ± 0.16 µL, excitation wavelength 976 nm, emission wavelength 800 ± 25 nm, exposure time 5000 ms. Image size 674 µm × 674 µm.

Figure S6. Annotated Figure S5. Blue circles marks the localization of UCNP-COOHs. Image size 674 µm × 674 µm.

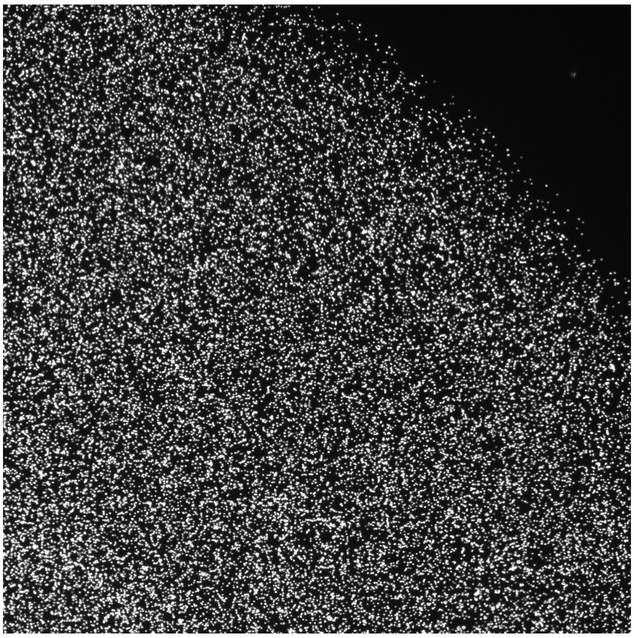

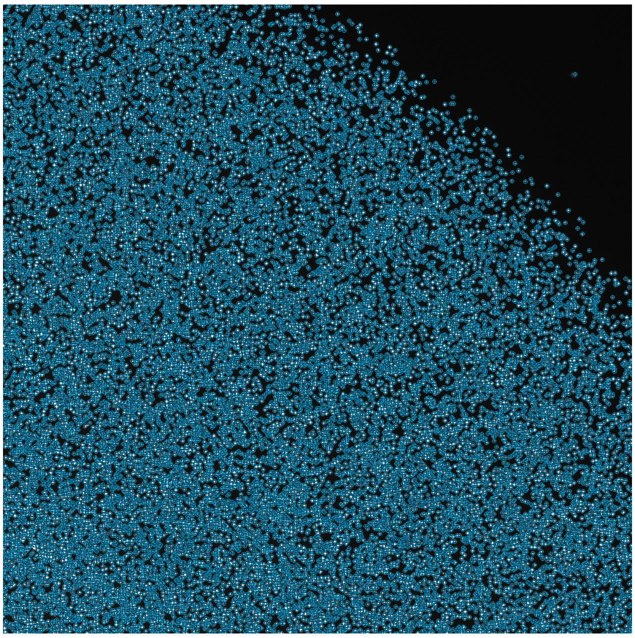

Figure S7. Epiphoton-upconversion microscopy. A part of a dried droplet close to the droplet edge, UCNP-COOH mass concentration 126 ng mL^{-1}, dispersion volume 2.46 ± 0.16 µL, excitation wavelength 976 nm, emission wavelength 800 ± 25 nm, exposure time 5000 ms. Image size 674 µm × 674 µm.

Figure S8. Annotated Figure S7. Blue circles marks the localization of UCNP-COOHs. Image size 674 µm × 674 µm.

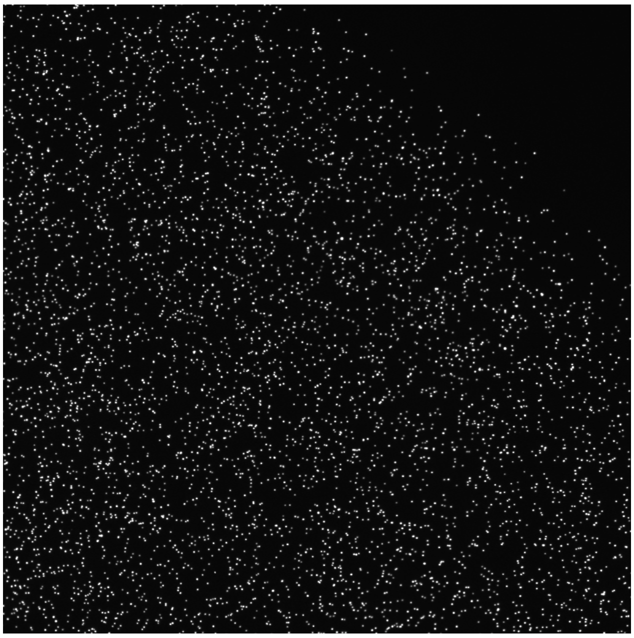

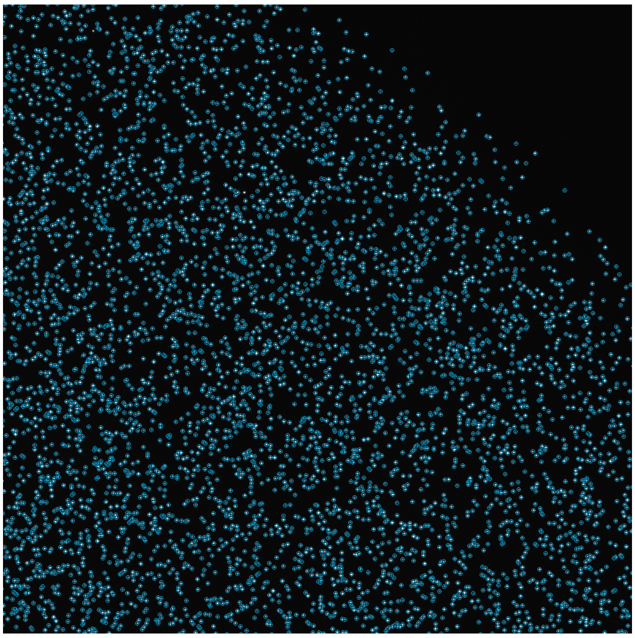

Figure S9. Epiphoton-upconversion microscopy. A part of a dried droplet close to the droplet edge, UCNP-COOH mass concentration 23 ng mL^{-1}, dispersion volume 2.46 ± 0.16 µL, excitation wavelength 976 nm, emission wavelength 800 ± 25 nm, exposure time 5000 ms. Image size 674 µm × 674 µm.

Figure S10. Annotated Figure S9. Blue circles marks the localization of UCNP-COOHs. Image size 674 µm × 674 µm.

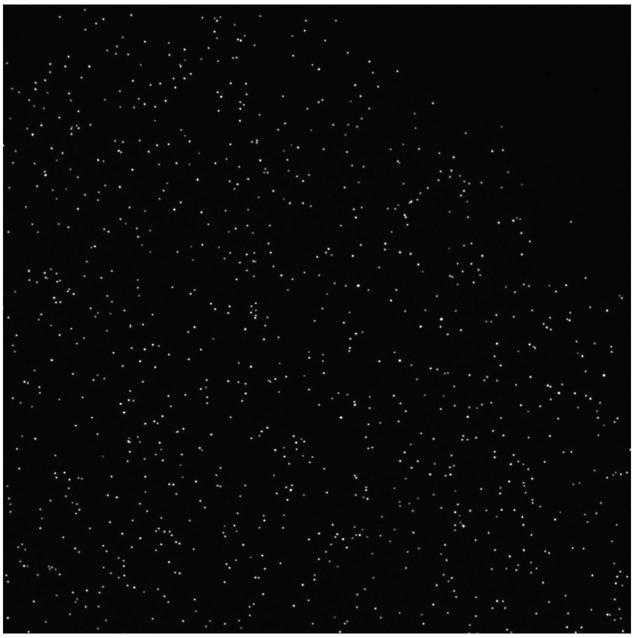

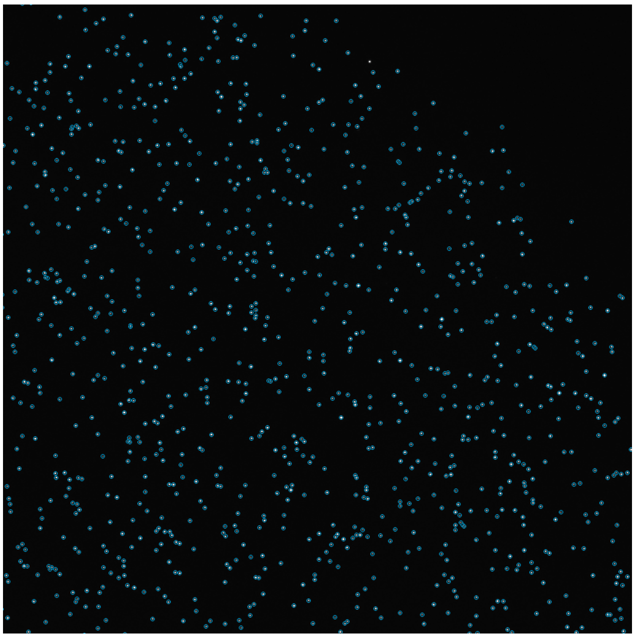

Figure S11. Epiphoton-upconversion microscopy. A part of a dried droplet close to the droplet edge, UCNP-COOH mass concentration 4.3 ng mL^{-1}, dispersion volume 2.46 ± 0.16 µL, excitation wavelength 976 nm, emission wavelength 800 ± 25 nm, exposure time 5000 ms. Image size 674 µm × 674 µm.

Figure S12. Annotated Figure S11. Blue circles marks the localization of UCNP-COOHs. Image size 674 µm × 674 µm.

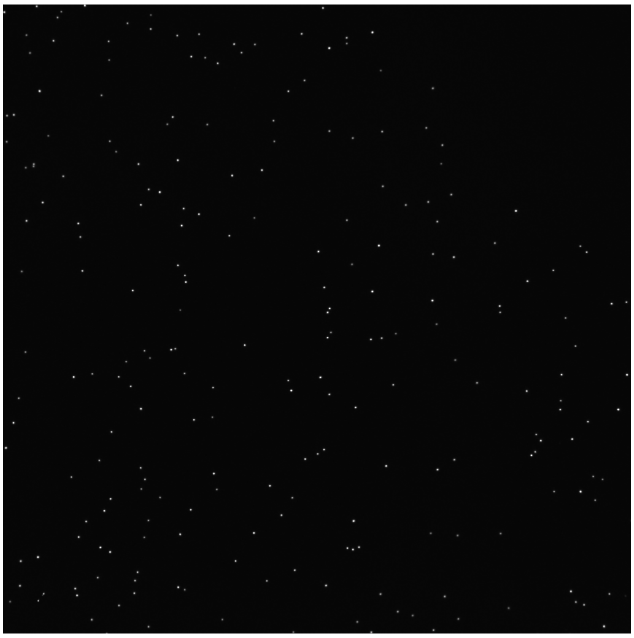

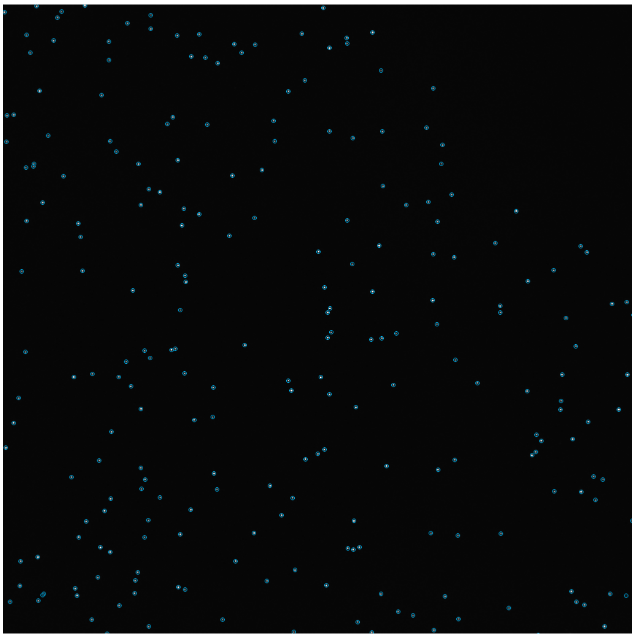

Figure S13. Epiphoton-upconversion microscopy. A part of a dried droplet close to the droplet edge, UCNP-COOH mass concentration 0.79 ng mL^{-1}, dispersion volume 2.46 ± 0.16 µL, excitation wavelength 976 nm, emission wavelength 800 ± 25 nm, exposure time 5000 ms. Image size 674 µm × 674 µm.

Figure S14. Annotated Figure S13. Blue circles marks the localization of UCNP-COOHs. Image size 674 µm × 674 µm.

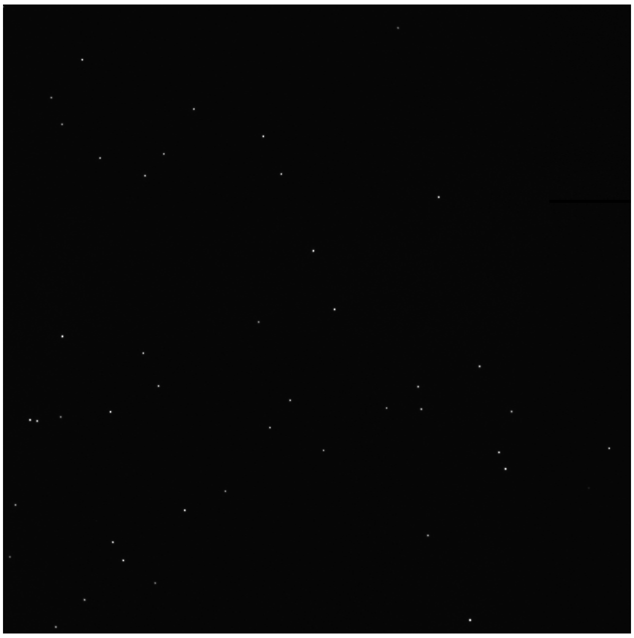

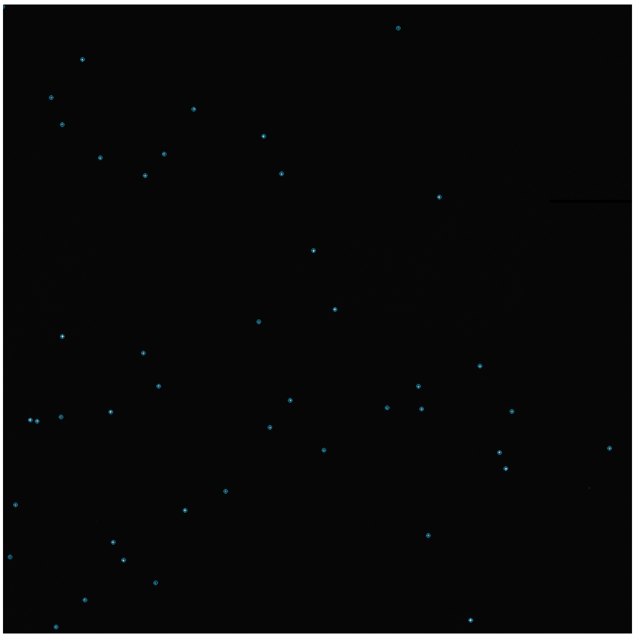

Figure S15. Epiphoton-upconversion microscopy. A part of a dried droplet close to the droplet edge, UCNP-COOH mass concentration 0.15 ng mL^{-1}, dispersion volume 2.46 ± 0.16 µL, excitation wavelength 976 nm, emission wavelength 800 ± 25 nm, exposure time 5000 ms. Image size 674 µm × 674 µm.

Figure S16. Annotated Figure S15. Blue circles marks the localization of UCNP-COOHs. Image size 674 µm × 674 µm.

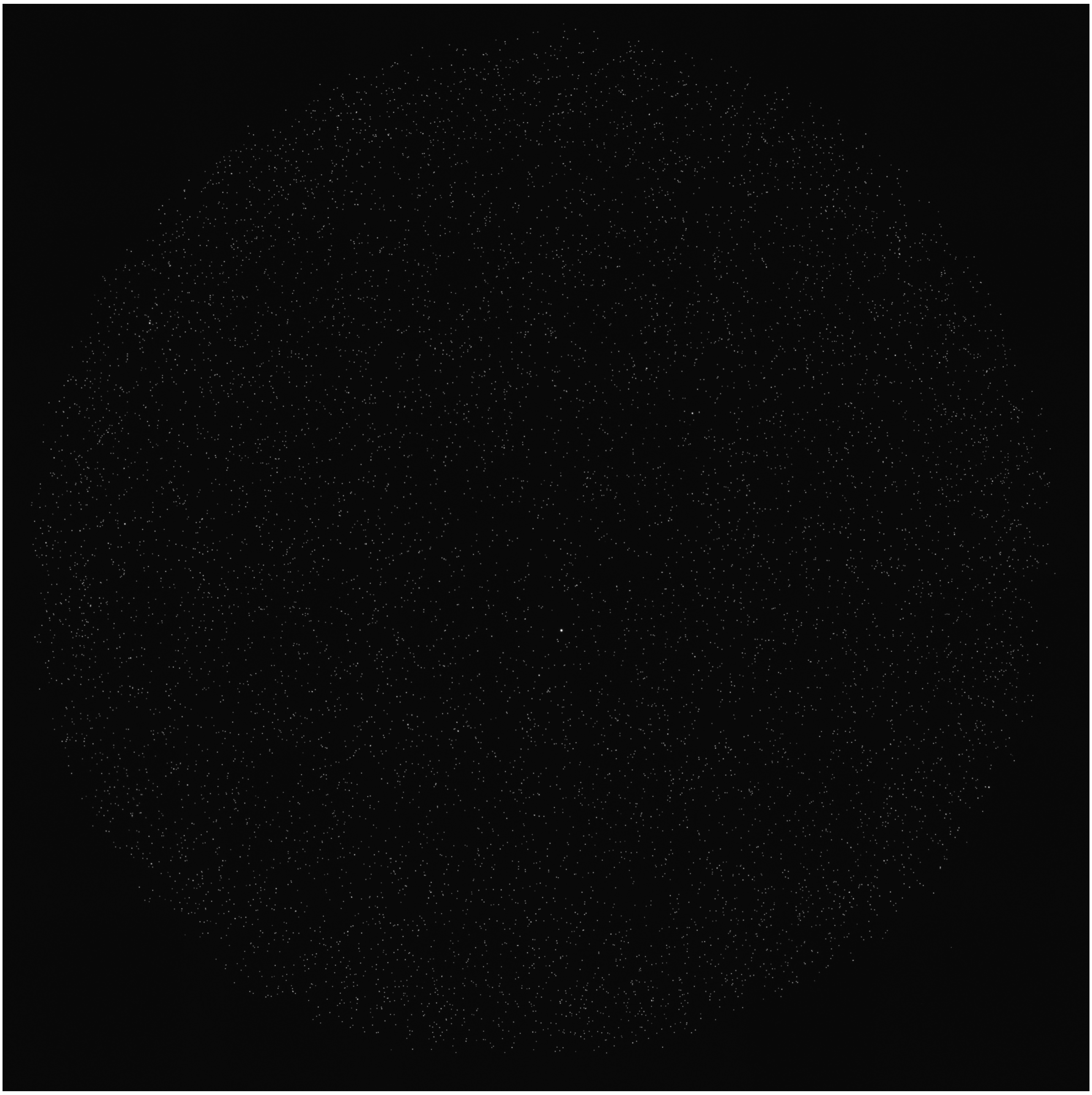

Figure S17. Epiphoton-upconversion microscopy. Dried droplet of UCNP-COOHs dispersion containing 45 mM Tris and 45 mM H_3BO_3. UCNP-COOH mass concentration ~3 ng mL^{-1}, droplet volume ~2.5 µL, excitation wavelength 976 nm, emission wavelength 800 ± 25 nm, exposure time 5000 ms. Image size 3116 µm × 3116 µm.

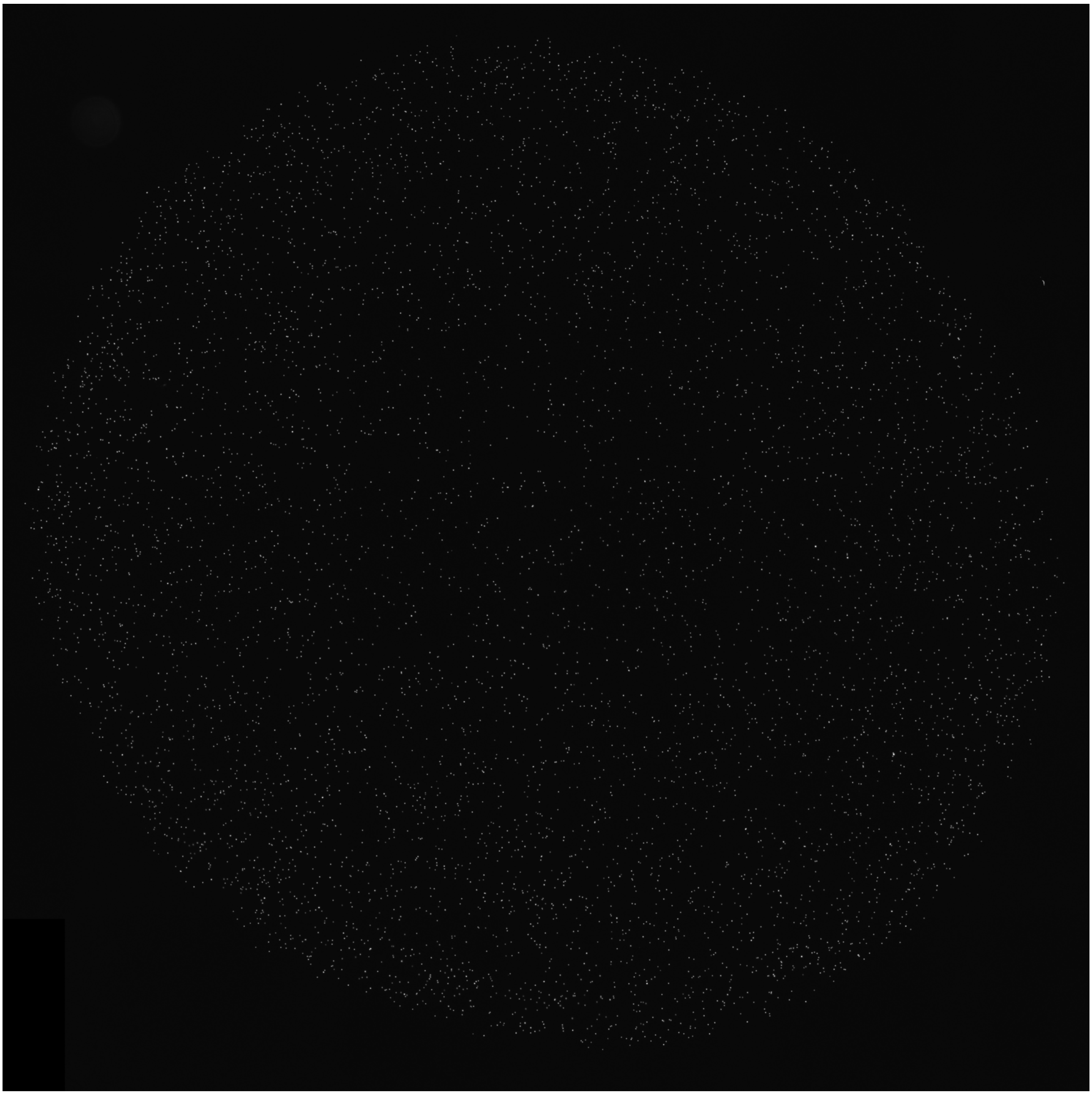

Figure S18. Epiphoton-upconversion microscopy. Dried droplet of UCNP-COOHs dispersion containing 10% (v/v) of bovine plasma. UCNP-COOH mass concentration ~3 ng mL^{-1}, droplet volume ~2.5 µL, excitation wavelength 976 nm, emission wavelength 800 ± 25 nm, exposure time 5000 ms. Image size 2527 µm × 2527 µm.

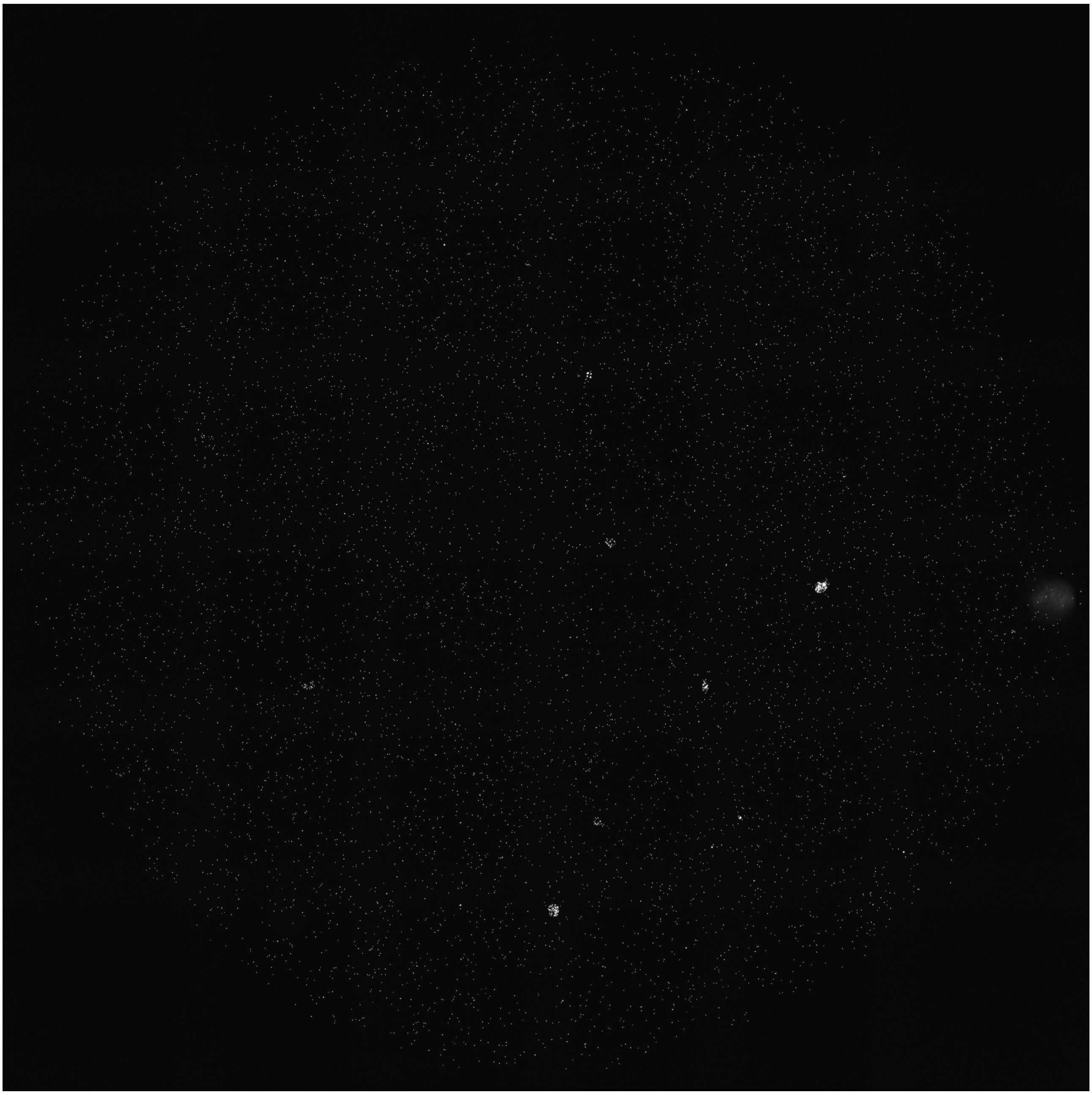

Figure S19. Epiphoton-upconversion microscopy. Dried droplet of UCNP-COOHs dispersion containing 0.36% (v/v) of orange nectar. UCNP-COOH mass concentration ~3 ng mL^{-1}, dispersion volume ~2.5 µL, excitation wavelength 976 nm, emission wavelength 800 ± 25 nm, exposure time 5000 ms. Image size 3116 µm × 3116 µm.

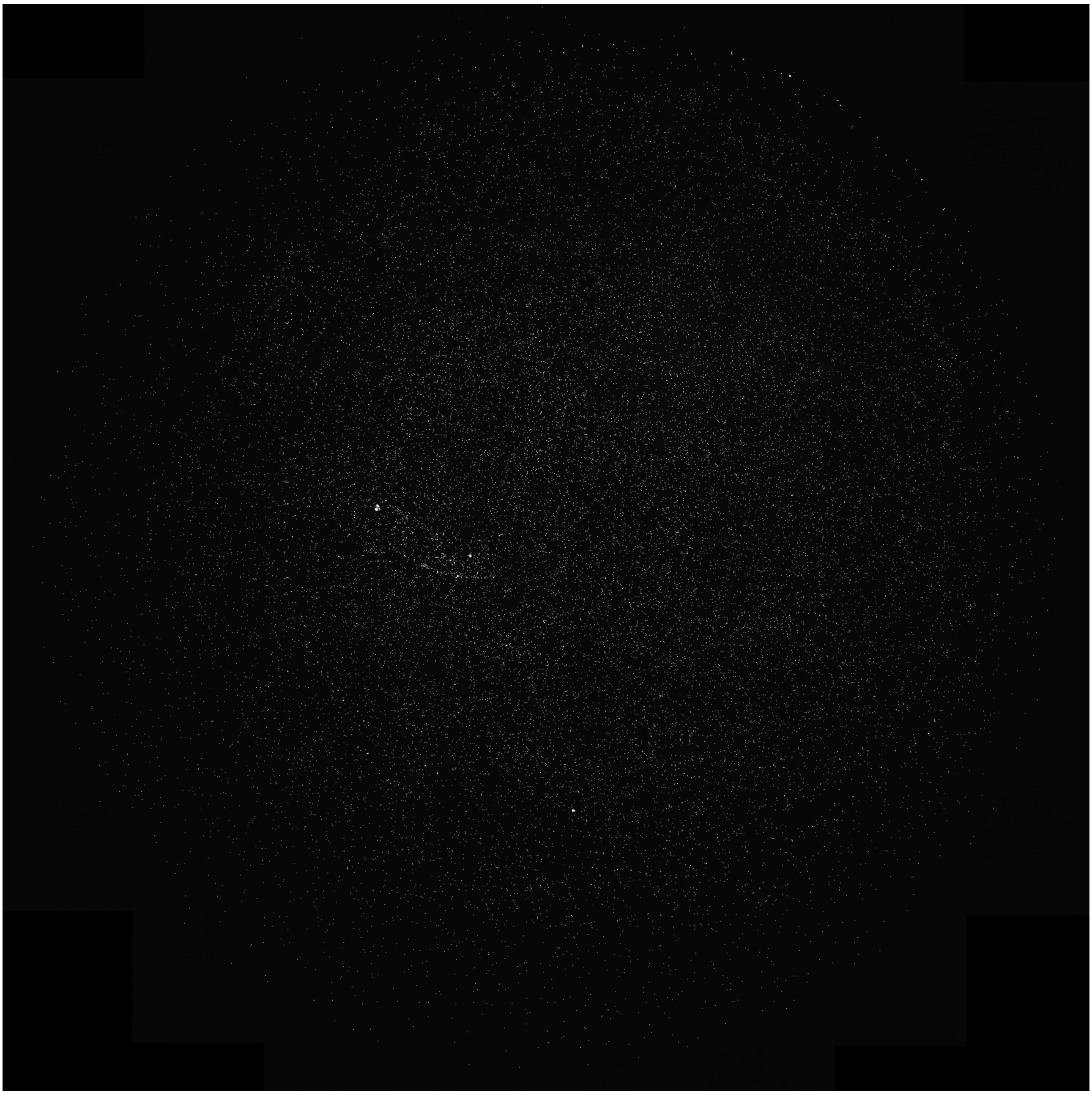

Figure S20. Epiphoton-upconversion microscopy. Dried droplet of UCNPs in a chloroform containing 0.05% (w/v) of polystyrene. UCNP mass concentration ~15 ng mL^{-1}, dispersion volume ~1 µL, excitation wavelength 976 nm, emission wavelength 800 ± 25 nm, exposure time 5000 ms. Image size 4127 µm × 4127 µm.

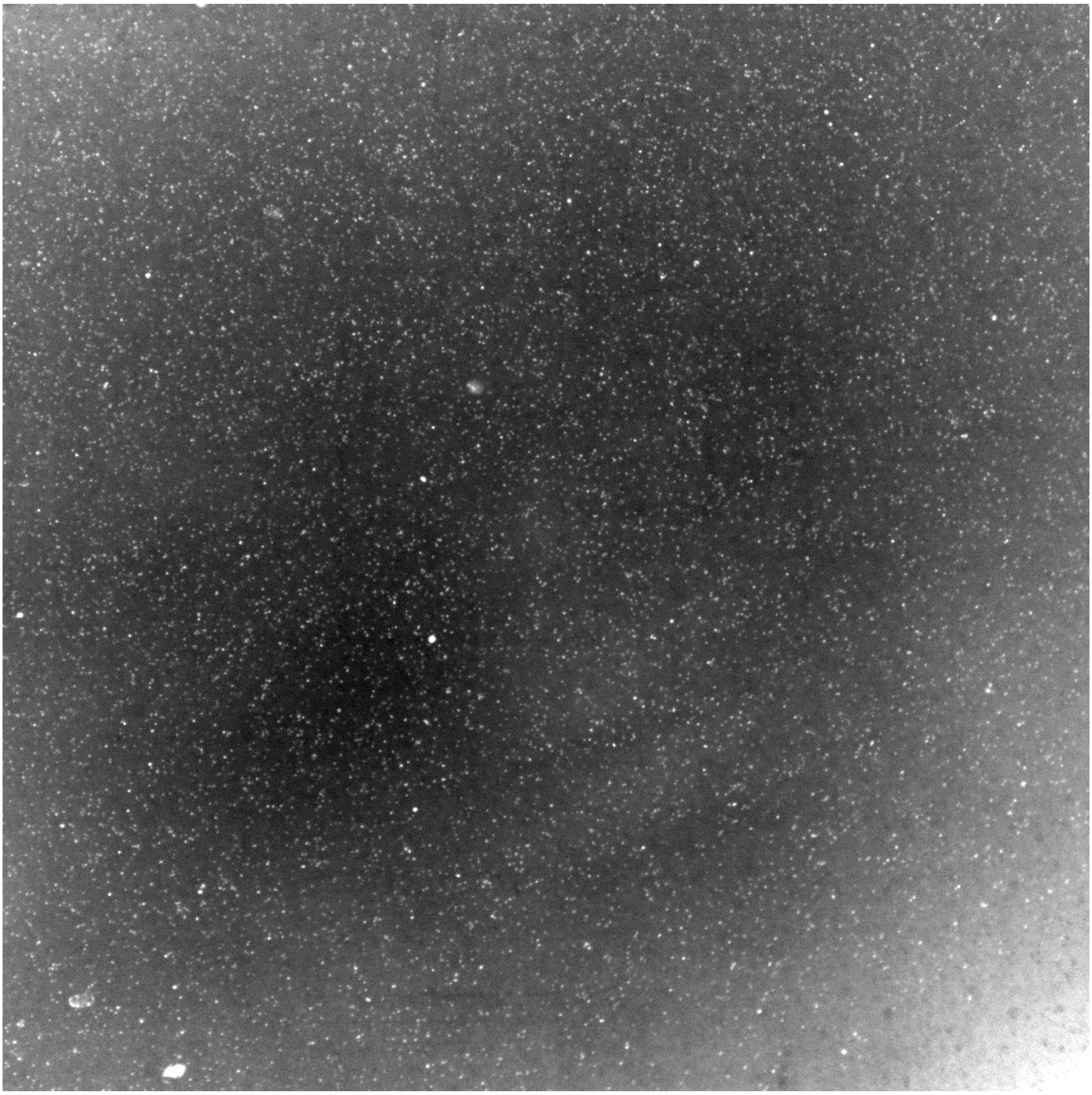

Figure S21. Epifluorescence microscopy. Epifluorescence microscopy of ~80 nm Nile red-doped polystyrene nanoparticles (excitation 520 nm, emission 650 ± 25 nm, exposure time 20 s, objective magnification 20×, numerical aperture 0.50). Image size 674 μm × 674 μm.

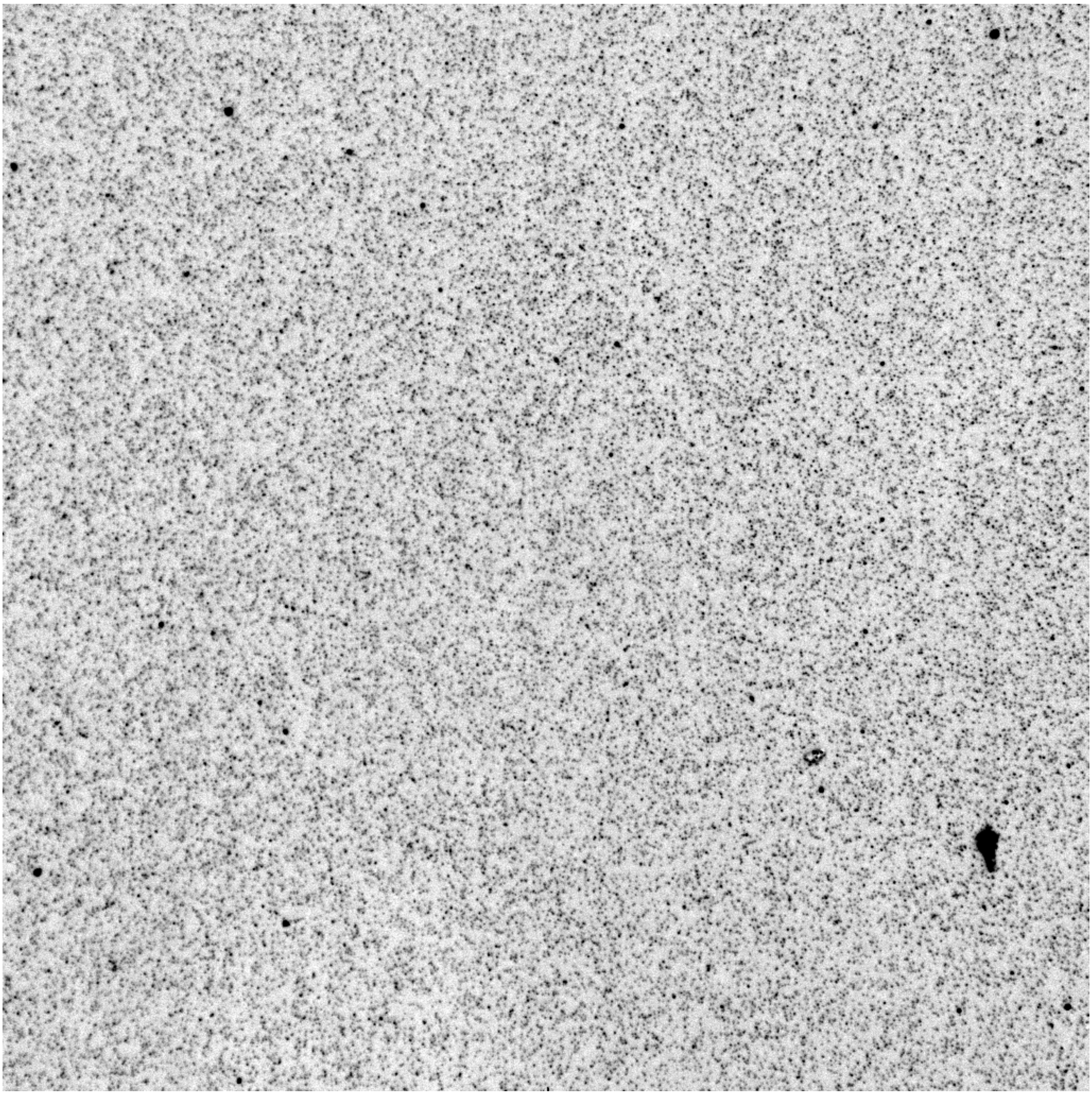

Figure S22. Bright-field microscopy. Bright-field microscopy of ~90 nm silver nanoparticles (wavelength 475 ± 25 nm, objective magnification 20×, numerical aperture 0.50, see Note S3 for the description of the exposure and correction scheme). Image size 674 µm × 674 µm.

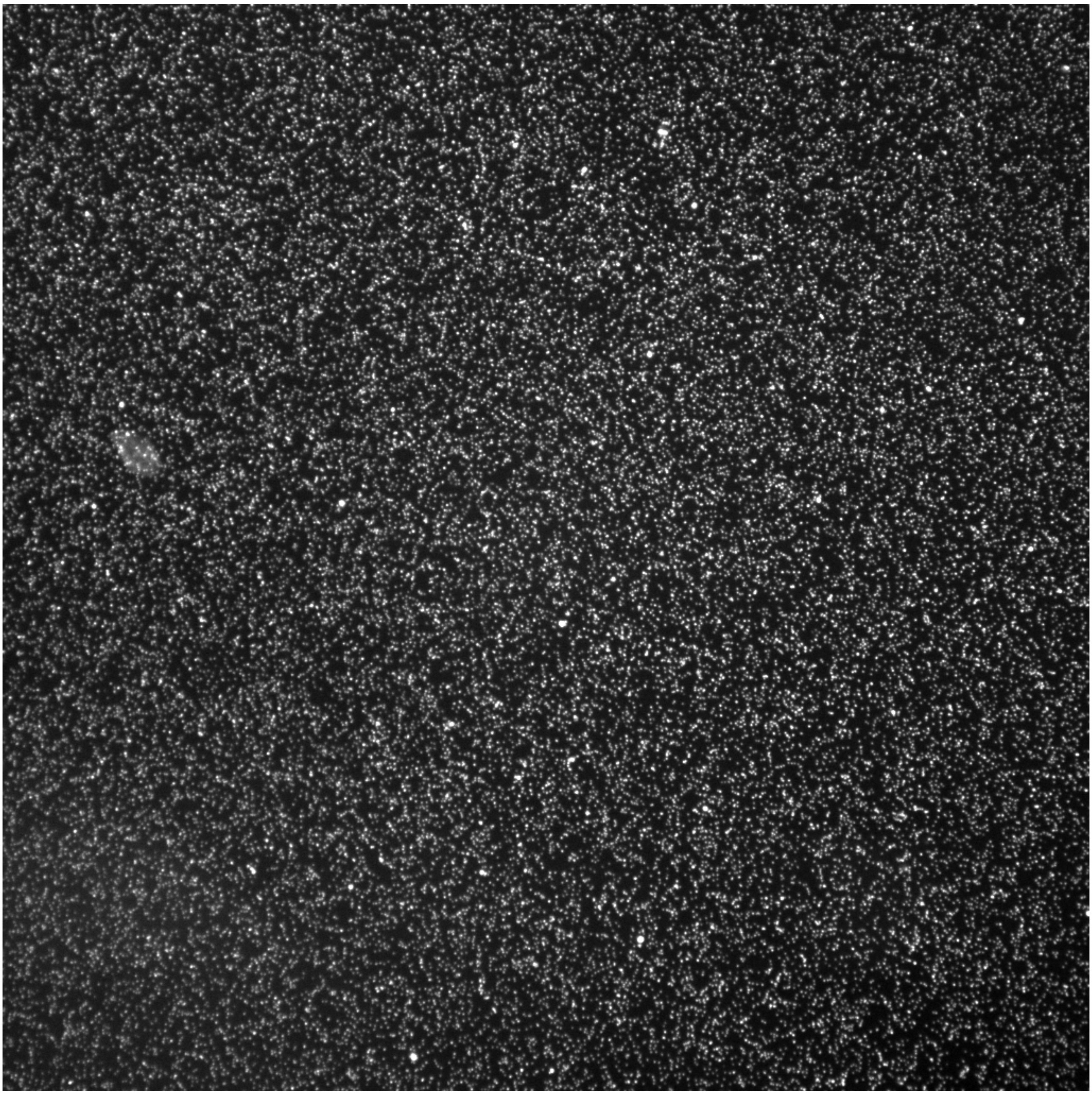

Figure S23. Dark-field microscopy. Dark-field microscopy of ~90 nm silver nanoparticles (wavelength 475 ± 25 nm, objective magnification 20×, numerical aperture 0.50, exposure time 2500 ms). Image size 674 µm × 674 µm.

References

(1) Hlaváček, A.; Farka, Z.; Mickert, M. J.; Kostiv, U.; Brandmeier, J. C.; Horák, D.; Skládal, P.; Foret, F.; Gorris, H. H. Bioconjugates of Photon-Upconversion Nanoparticles for Cancer Biomarker Detection and Imaging. *Nat. Protoc.* **2022**, *17* (4), 1028–1072. https://doi.org/10.1038/s41596-021-00670-7.

(2) Srivastava, P.; Tavernaro, I.; Genger, C.; Welker, P.; Hübner, O.; Resch-Genger, U. Multicolor Polystyrene Nanosensors for the Monitoring of Acidic, Neutral, and Basic pH Values and Cellular Uptake Studies. *Anal. Chem.* **2022**, *94* (27), 9656–9664. https://doi.org/10.1021/acs.analchem.2c00944.

(3) Bastús, N. G.; Merkoçi, F.; Piella, J.; Puntes, V. Synthesis of Highly Monodisperse Citrate-Stabilized Silver Nanoparticles of up to 200 nm: Kinetic Control and Catalytic Properties. *Chem. Mater.* **2014**, *26* (9), 2836–2846. https://doi.org/10.1021/cm500316k.

(4) Materials Data on NaYF4 by Materials Project, 2020. https://doi.org/10.17188/1207335.

(5) Kimoto, S.; Dick, W. D.; Hunt, B.; Szymanski, W. W.; McMurry, P. H.; Roberts, D. L.; Pui, D. Y. H. Characterization of Nanosized Silica Size Standards. *Aerosol Sci. Technol.* **2017**, *51* (8), 936–945. https://doi.org/10.1080/02786826.2017.1335388.

(6) Mackenzie, L. E.; Goode, J. A.; Vakurov, A.; Nampi, P. P.; Saha, S.; Jose, G.; Millner, P. A. The Theoretical Molecular Weight of NaYF4:RE Upconversion Nanoparticles. *Sci. Rep.* **2018**, *8* (1), 1106. https://doi.org/10.1038/s41598-018-19415-w.

(7) Hlaváček, A.; Křivánková, J.; Brožková, H.; Weisová, J.; Pizúrová, N.; Foret, F. Absolute Counting Method with Multiplexing Capability for Estimating the Number Concentration of Nanoparticles Using Anisotropically Collapsed Gels. *Anal. Chem.* **2022**, *94* (41), 14340–14348. https://doi.org/10.1021/acs.analchem.2c02989.

(8) Vo, N. T.; Atwood, R. C.; Drakopoulos, M. Radial Lens Distortion Correction with Sub-Pixel Accuracy for X-Ray Micro-Tomography. *Opt. Express* **2015**, *23* (25), 32859–32868. https://doi.org/10.1364/OE.23.032859.

(9) Schindelin, J.; Arganda-Carreras, I.; Frise, E.; Kaynig, V.; Longair, M.; Pietzsch, T.; Preibisch, S.; Rueden, C.; Saalfeld, S.; Schmid, B.; Tinevez, J.-Y.; White, D. J.; Hartenstein, V.; Eliceiri, K.; Tomancak, P.; Cardona, A. Fiji: An Open-Source Platform for Biological-Image Analysis. *Nat. Methods* **2012**, *9* (7), 676–682. https://doi.org/10.1038/nmeth.2019.

(10) Preibisch, S.; Saalfeld, S.; Tomancak, P. Globally Optimal Stitching of Tiled 3D Microscopic Image Acquisitions. *Bioinformatics* **2009**, *25* (11), 1463–1465. https://doi.org/10.1093/bioinformatics/btp184.

(11) BIPM, IEC, IFCC, ILAC, ISO, IUPAC, IUPAP, and OIML. *Guide to the Expression of Uncertainty in Measurement” — Propagation of Distributions Using a Monte Carlo Method*; Joint Committee for Guides in Metrology, JCGM 101:2008, 2008.

(12) Speiser, A.; Müller, L.-R.; Hoess, P.; Matti, U.; Obara, C. J.; Legant, W. R.; Kreshuk, A.; Macke, J. H.; Ries, J.; Turaga, S. C. Deep Learning Enables Fast and Dense Single-Molecule Localization with High Accuracy. *Nat. Methods* **2021**, *18* (9), 1082–1090. https://doi.org/10.1038/s41592-021-01236-x.

(13) Etheridge, T. J.; Carr, A. M.; Herbert, A. D. GDSC SMLM: Single-Molecule Localisation Microscopy Software for ImageJ. *Wellcome Open Res.* **2022**, *7*. https://doi.org/10.12688/wellcomeopenres.18327.1.

(14) Torquato, S.; Lu, B.; Rubinstein, J. Nearest-Neighbour Distribution Function for Systems on Interacting Particles. *J. Phys. Math. Gen.* **1990**, *23* (3), L103. https://doi.org/10.1088/0305-4470/23/3/005.

(15) *Water Density Table*. https://www.internetchemistry.com/chemical-data/water-density-table.php (accessed 2024-10-01).

(16) Lahtinen, S.; Lyytikäinen, A.; Päkkilä, H.; Hömppi, E.; Perälä, N.; Lastusaari, M.; Soukka, T. Disintegration of Hexagonal NaYF4:Yb3+,Er3+ Upconverting Nanoparticles in Aqueous Media: The Role of Fluoride in Solubility Equilibrium. *J. Phys. Chem. C* **2017**, *121* (1), 656–665. https://doi.org/10.1021/acs.jpcc.6b09301.

(17) Batista, E.; Pinto, L.; Filipe, E.; van der Veen, A. M. H. Calibration of Micropipettes: Test Methods and Uncertainty Analysis. *Measurement* **2007**, *40* (3), 338–342. https://doi.org/10.1016/j.measurement.2006.05.012.

(18) Möckl, L.; Roy, A. R.; Moerner, W. E. Deep Learning in Single-Molecule Microscopy: Fundamentals, Caveats, and Recent Developments. *Biomed. Opt. Express* **2020**, *11* (3), 1633–1661. https://doi.org/10.1364/BOE.386361.

(19) Peters, R.; Herrera-Rivera, Z.; Undas, A.; Lee, M. van der; Marvin, H.; Bouwmeester, H.; Weigel, S. Single Particle ICP-MS Combined with a Data Evaluation Tool as a Routine Technique for the Analysis of Nanoparticles in Complex Matrices. *J. Anal. At. Spectrom.* **2015**, *30* (6), 1274–1285. https://doi.org/10.1039/C4JA00357H.

(20) Zhang, C.; Johnson, L. W. Simple and Accurate Quantification of Quantum Dots via Single-Particle Counting. *J. Am. Chem. Soc.* **2008**, *130* (12), 3750–3751. https://doi.org/10.1021/ja711493q.

(21) Zhu, S.; Yang, L.; Long, Y.; Gao, M.; Huang, T.; Hang, W.; Yan, X. Size Differentiation and Absolute Quantification of Gold Nanoparticles via Single Particle Detection with a Laboratory-Built High-Sensitivity Flow Cytometer. *J. Am. Chem. Soc.* **2010**, *132* (35), 12176–12178. https://doi.org/10.1021/ja104052c.

(22) Lan, W.-J.; Holden, D. A.; Zhang, B.; White, H. S. Nanoparticle Transport in Conical-Shaped Nanopores. *Anal. Chem.* **2011**, *83* (10), 3840–3847. https://doi.org/10.1021/ac200312n.

(23) Tian, Y.; Tian, D.; Peng, X.; Qiu, H. Critical Parameters to Standardize the Size and Concentration Determination of Nanomaterials by Nanoparticle Tracking Analysis. *Int. J. Pharm.* **2024**, *656*, 124097. https://doi.org/10.1016/j.ijpharm.2024.124097.

(24) Tai, L.-A.; Kang, Y.-T.; Chen, Y.-C.; Wang, Y.-C.; Wang, Y.-J.; Wu, Y.-T.; Liu, K.-L.; Wang, C.-Y.; Ko, Y.-F.; Chen, C.-Y.; Huang, N.-C.; Chen, J.-K.; Hsieh, Y.-F.; Yew, T.-R.; Yang, C.-S. Quantitative Characterization of Nanoparticles in Blood by Transmission Electron Microscopy with a Window-Type Microchip Nanopipet. *Anal. Chem.* **2012**, *84* (15), 6312–6316. https://doi.org/10.1021/ac301523n.
